# A Least Absolute Shrinkage and Selection Operator-Derived Predictive Model for Postoperative Respiratory Failure in a Heterogeneous Adult Elective Surgery Patient Population

**DOI:** 10.1016/j.chstcc.2023.100025

**Published:** 2023-10-20

**Authors:** Jacqueline C. Stocking, Sandra L. Taylor, Sili Fan, Theodora Wingert, Christiana Drake, J. Matthew Aldrich, Michael K. Ong, Alpesh N. Amin, Rebecca A. Marmor, Laura Godat, Maxime Cannesson, Michael A. Gropper, Garth H. Utter, Christian E. Sandrock, Christian Bime, Jarrod Mosier, Vignesh Subbian, Jason Y. Adams, Nicholas J. Kenyon, Timothy E. Albertson, Joe G. N. Garcia, Ivo Abraham

**Affiliations:** Division of Pulmonary, Critical Care and Sleep Medicine (J. C. S., C. E. S., J. Y. A., N. J. K., and T. E. A.), Department of Internal Medicine, the Department of Public Health Sciences (S. L. T. and S. F.), the Outcomes Research Group (G. H. U.), Department of Surgery, University of California Davis, Sacramento, the Department of Anesthesiology and Perioperative Medicine (T. W. and M. C.), University of California Los Angeles, the Department of Medicine (M. K. O.), University of California Los Angeles, the VA Greater Los Angeles Healthcare System (M. K. O.), Los Angeles, the Department of Statistics (C. D.), University of California Davis, Davis, the Department of Anesthesia and Perioperative Care (J. M. A. and M. A. G.), University of California, San Francisco, San Francisco, the Department of Medicine (A. N. A.), University of California Irvine, Irvine, the Department of Surgery (R. A. M. and L. G.), University of California San Diego, San Diego, the College of Medicine (C. B. and J. M.), University of Arizona Health Sciences, the Department of Biomedical Engineering (V. S.), College of Engineering, the Center for Health Outcomes and PharmacoEconomic Research (I. A.), University of Arizona, Tucson, AZ, and The University of Florida-Scripps Research Institute (J. G. N. G.), Jupiter, FL.

**Keywords:** bootstrapping, least absolute shrinkage and selection operator, phenotyping, postoperative, predictive model, respiratory failure

## Abstract

**BACKGROUND:**

Postoperative respiratory failure (PRF) is associated with increased hospital charges and worse patient outcomes. Reliable prediction models can help to guide postoperative planning to optimize care, to guide resource allocation, and to foster shared decision-making with patients.

**RESEARCH QUESTION:**

Can a predictive model be developed to accurately identify patients at high risk of PRF?

**STUDY DESIGN AND METHODS:**

In this single-site proof-of-concept study, we used structured query language to extract, transform, and load electronic health record data from 23,999 consecutive adult patients admitted for elective surgery (2014–2021). Our primary outcome was PRF, defined as mechanical ventilation after surgery of > 48 h. Predictors of interest included demographics, comorbidities, and intraoperative factors. We used logistic regression to build a predictive model and the least absolute shrinkage and selection operator procedure to select variables and to estimate model coefficients. We evaluated model performance using optimism-corrected area under the receiver operating curve and area under the precision-recall curve and calculated sensitivity, specificity, positive and negative predictive values, and Brier scores.

**RESULTS:**

Two hundred twenty-five patients (0.94%) demonstrated PRF. The 18-variable predictive model included: operations on the cardiovascular, nervous, digestive, urinary, or musculoskeletal system; surgical specialty orthopedic (nonspine); Medicare or Medicaid (as the primary payer); race unknown; American Society of Anesthesiologists class ≥ III; BMI of 30 to 34.9 kg/m^2^; anesthesia duration (per hour); net fluid at end of the operation (per liter); median intraoperative FIO_2_, end title CO_2_, heart rate, and tidal volume; and intraoperative vasopressor medications. The optimism-corrected area under the receiver operating curve was 0.835 (95% CI,0.808–0.862) and the area under the precision-recall curve was 0.156 (95% CI, 0.105–0.203).

**INTERPRETATION:**

This single-center proof-of-concept study demonstrated that a structured query language extract, transform, and load process, based on readily available patient and intraoperative variables, can be used to develop a prediction model for PRF. This PRF prediction model is scalable for multicenter research. Clinical applications include decision support to guide postoperative level of care admission and treatment decisions.

Postoperative respiratory failure (PRF), defined as requiring mechanical ventilation (MV) after surgery of > 48 h, is a major source of morbidity.^1^ With an incidence of 0.2% to 7.5%,^1–4^ PRF is associated with increased hospital charges, hospital and ICU lengths of stay, and in-hospital and postdischarge morbidity and mortality.^5–8^ Risk factors for PRF in patients undergoing a broad spectrum of surgical procedures have been analyzed in prior predictive models.^1,9,10^

However, consensus among these models is lacking because of differences in PRF definition, population, and predictors of interest. Other studies have focused on homogeneous patient populations, such as abdominal,^11^ neurological,^12^ or cardiovascular^13^ surgery patients, often including both elective and emergent surgical procedures. The Centers for Medicare & Medicaid Services includes PRF that occurs after elective surgery in the Hospital-Acquired Condition Reduction and Hospital Compare Public Reporting Programs, yet progress in reducing the incidence of PRF has been hindered by this lack of consensus in identifying the most at-risk patients. Identifying patients at increased risk of PRF after elective surgery is an important step toward developing clinical workflows to improve postoperative care and outcomes while appropriately allocating hospital resources. Such workflows include postoperative level of care, admission location, monitoring, and treatment orders for at-risk patients.

Herein we describe an automated structured query language (SQL)-based extract, transform, and load (ETL) procedure that enables rapid acquisition of data exclusively from an electronic health record (EHR). We then used the selected and validated data to develop a single-site proof-of-concept predictive model^14^ for PRF after elective surgery in adults. Our aim was to develop a model that considered a patient’s pre-existing risk factors, intraoperative care and physiologic parameters, and status on exiting the operating room to identify patients at risk of PRF. We hypothesized that our model would have at least good discrimination and would be well calibrated across its range of predicted probabilities. Our methods will allow us to expand our SQL ETL process across the five centers of our University of California Critical Care Research Collaborative for further model development and validation. Generating standardized, automated approaches to large-scale multicenter research using real-world data is crucial in predictive modeling of rare adverse events, such as PRF.

## Study Design and Methods

This retrospective cohort study was approved by the institutional review board at the University of California, Davis; the requirement for informed consent was waived. This article adheres to the Strengthening the Reporting of Observational Studies in Epidemiology Statement^15^ and the Transparent Reporting of a Multivariable Prediction Model for Individual Prognosis Or Diagnosis^16^ guidelines.

### Study Design, Setting, and Population

We analyzed 23,999 consecutive adult patients undergoing elective surgery at a single academic center (2014–2021). The start date was selected based on the conversion from paper to EHR clinical documentation for perioperative services and the end date was selected to provide access to 8 full calendar years of data for ETL. Inclusion criteria were adults aged 18 years and older, elective surgical admissions, undergoing an operation within 24 h of admission, and general anesthesia. Exclusion criteria were transfers from another hospital and a tracheostomy present on admission. The primary outcome was PRF. Secondary outcomes included hospital and ICU length of stay and discharge disposition.

### Data ETL Procedure

PRF was defined as > 48 h of MV, from the anesthesia end time to hospital discharge. Predictors of interest spanned the preoperative and intraoperative care continuum and included demographics, pre-existing comorbidities, and preoperative and intraoperative factors (e-Table 1). We used SQL coding to perform the data ETL procedure from our Epic EHR (e-Appendix 1). Two clinicians validated data acquisition by comparing ETL output for 100% of patients with PRF and a random 10% of patients without PRF via manual independent chart review until agreement reached 100%. All variables had < 2.5% missing data; missingness was imputed to the cohort mode for categorical variables and median for continuous variables. Although other studies have included preoperative laboratory values, despite also having > 50% missing data^17^ and emergency surgery^18–20^ in their models, we opted not to include either. Although our health system, like others, has used an SQL ETL process for clinical data, this was our first use of this method for perioperative flow sheet data from the Epic OpTime module.

### Descriptive Statistics

We report the median and interquartile range for continuous variables and total number and percentage for categorical variables. We used Pearson’s χ^2^ test and the Wilcoxon rank-sum test to compare patients with PRF with patients without PRF for categorical and continuous variables, respectively. Significance was set a priori at *P* < .05. Data were analyzed using Stata MP version 18 software (StataCorp) and R version 4.2.2 software (R Foundation for Statistical Computing).

### Predictive Model Development and Evaluation

**Figure 1 –:**
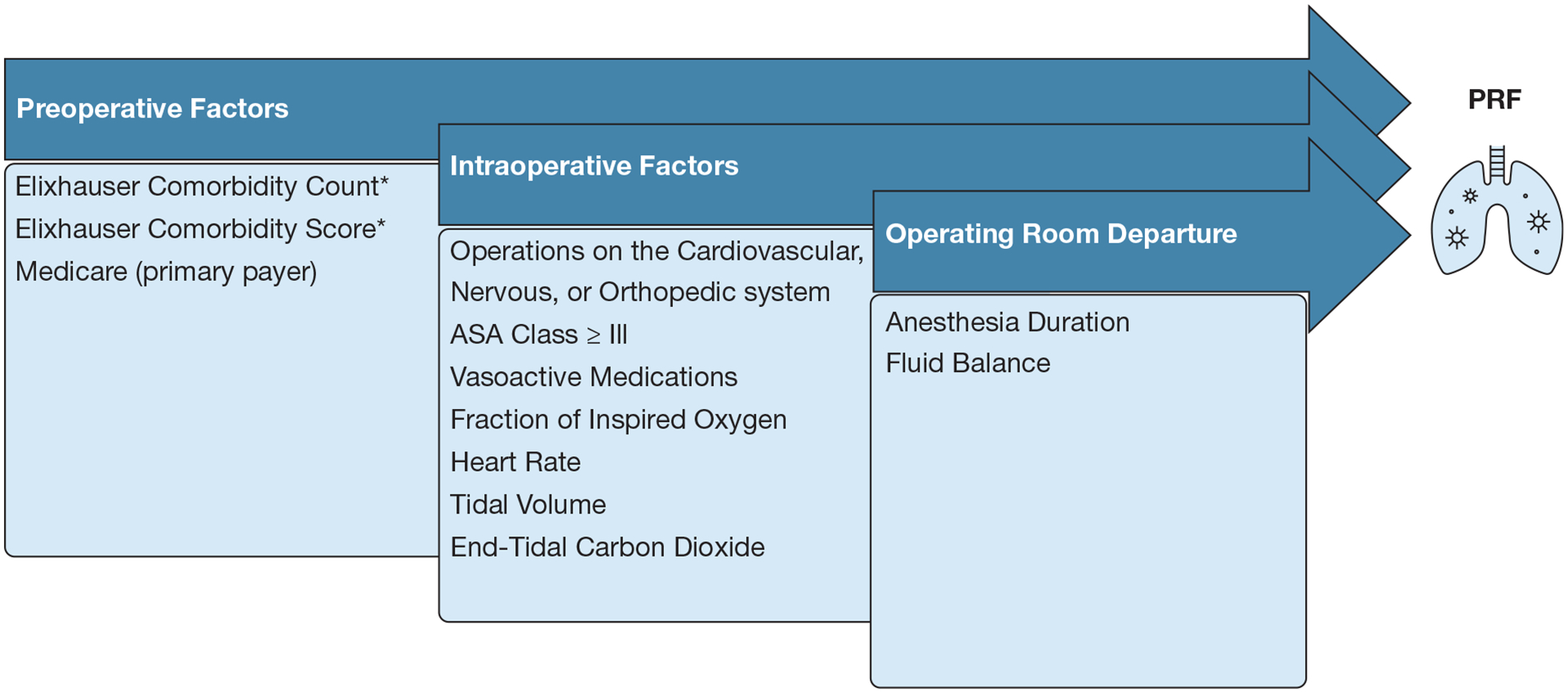
Conceptual framework of predictive model for postoperative respiratory failure. ASA = American Society of Anesthesiologists; PRF = postoperative respiratory failure. *Comorbid conditions included in the Elixhauser: congestive heart failure, cardiac arrythmias, valvular disease, pulmonary circulatory disorders, peripheral vascular disorders, hypertension (uncomplicated), hypertension (complicated), paralysis, other neurological disorders, chronic pulmonary disease, diabetes (uncomplicated), diabetes (complicated), hypothyroidism, renal failure, liver disease, peptic ulcer disease excluding bleeding, AIDS/HIV, lymphoma, metastatic cancer, solid tumor without metastasis, rheumatoid arthritis/collagen vascular diseases coagulopathy, obesity, weight loss , fluid and electrolyte disorders, blood loss anemia, deficiency anemia, alcohol abuse, drug abuse, psychoses, depression.^25^ Elixhauser comorbidity score is calculated by assigning weights to each comorbidity based on van Walraven et al.26 NOTE: Elixhauser was only used in e-Appendix 3 sensitivity analysis.

We used logistic regression to build the predictive model^14^ and least absolute shrinkage and selection operator (LASSO)^21^ regularization to select variables and estimate model coefficients (e-Table 2). Our conceptual model for the analysis considered a patient’s pre-existing risk factors, intraoperative factors, and status on exiting the operating room to identify patients at risk of PRF (Fig 1).

**Figure 2 –:**
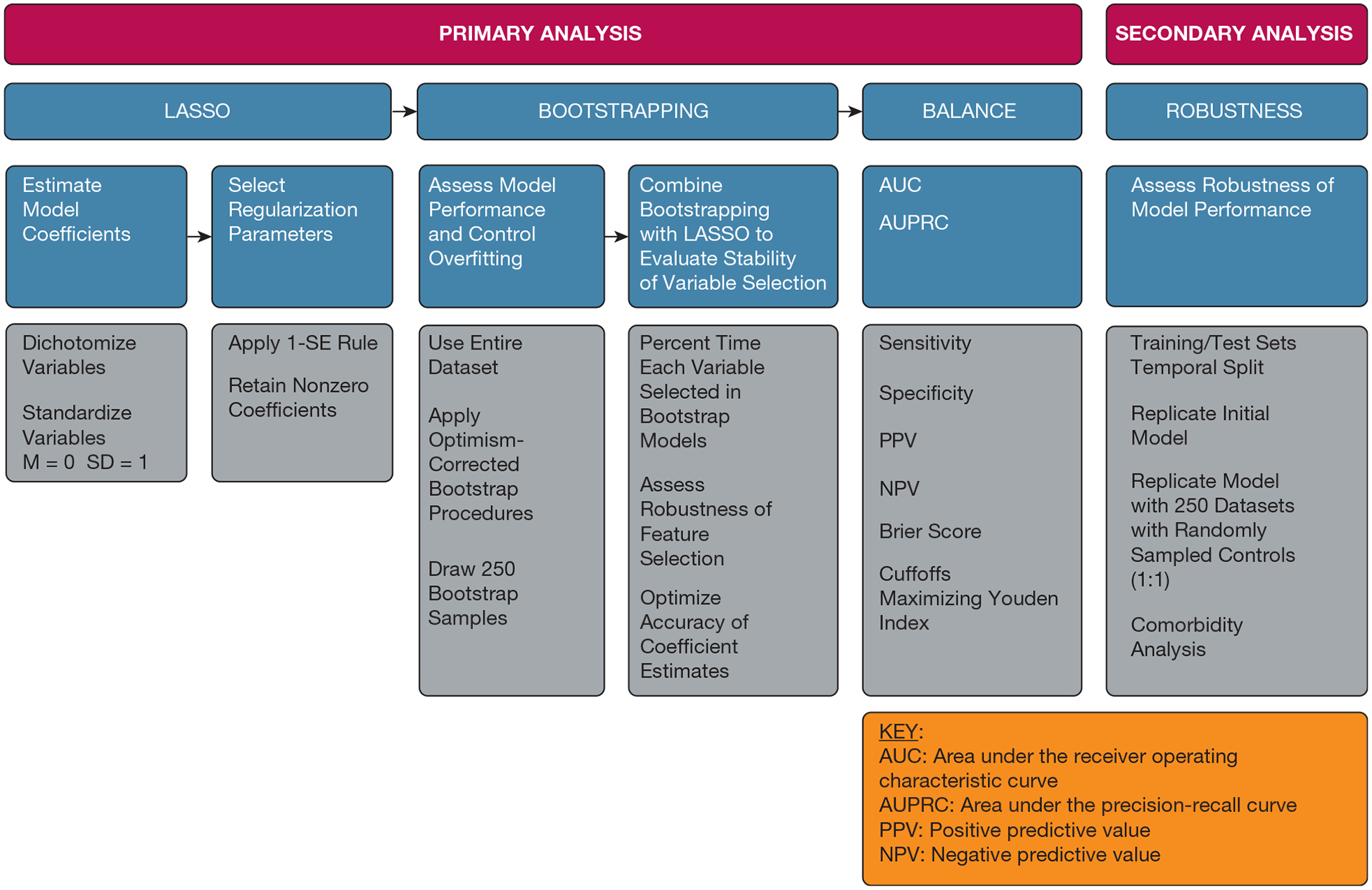
Diagram showing steps in the model derivation and validation process. M = mean; LASSO = least absolute shrinkage and selection operator.

Before model fitting, we dichotomized all categorical variables and standardized all numeric variables to have a mean of 0 and an SD of 1. To select the regularization parameter in the logistic LASSO model, we used a 10-fold cross-validation procedure and application of the 1-SE rule. This helps to ensure the generalizability and interpretability of the model by encouraging parsimony.^22^ We retained variables with nonzero coefficients from the fitted logistic LASSO model in the final prediction model. Given the small number of patients with PRF and the need to develop a model representative of the real-world prevalence of PRF, we used the entire data set in model development. To evaluate the performance of the model while controlling for overfitting, we used an optimism-corrected bootstrap procedure.^23^ We drew 250 bootstrap samples from the training data stratified by PRF group, maintaining the overall sample prevalence, and repeated the logistic LASSO modeling procedure on each bootstrap sample. We estimated optimism-corrected performance using the bootstrap models following Steyerberg.^23^ We additionally used a bootstrap procedure in combination with the logistic LASSO^24^ model fitting procedure to evaluate the stability of the variable selection procedure by calculating the frequency at which each variable was selected in the bootstrap models. This approach has the advantage of providing a robust feature selection performance and a more accurate estimate of coefficients. By training multiple LASSO models on different bootstrap samples of data, this method accounts for data variability and helps to identify features that consistently are important across different samples. We evaluated model performance using area under the receiver operating characteristic curve (AUC) and area under the precision-recall curve (AUPRC). Sensitivity, specificity, positive and negative predictive values, and Brier scores were calculated using a cutoff that maximized Youden’s index (Fig 2).

### Sensitivity and Robustness Analyses

We conducted secondary analyses to verify the optimism-corrected bootstrap procedure results and to evaluate robustly the model’s performance. For these analyses, data were split temporally into a training set (2014–2018) and a test set (2019–2021). First, the training set was used to develop a model in the same manner as the primary analysis and was evaluated on the test set. Second, again using the training set, we developed models using 1,000 bootstrapped data sets with equal numbers of patients with PRF and patients without PRF by randomly sampling from among patients without PRF. These models also were evaluated on the test set (e-Appendix 2). We also conducted a sensitivity analysis to determine the effect of the Elixhauser comorbidity count and score on model performance (e-Appendix 3).

## Results

### Pre-existing Patient and Intraoperative Characteristics

After 23,999 consecutive surgical encounters, PRF developed in 225 patients (0.94%). Patients with PRF were older, male, covered by Medicare, not obese, and admitted with multiple comorbidities (Table 1).^25,26^ Patients with PRF underwent longer anesthesia and surgery durations and more often underwent surgery on the cardiovascular system (Table 2). Patients with PRF also showed lower operative tidal volume and greater net positive fluid balance at the end of surgery and 24 h after surgery. Patients with PRF received more morphine equivalent units and more often received vasopressor medications. The most frequently used vasopressor medication in patients with PRF was norepinephrine and in patients without PRF was phenylephrine. Among all patients, the first oxygen device outside of the operating room was supplemental oxygen (47.3%), followed by room air (45.9%), MV (4.9%), noninvasive positive pressure ventilation (0.8%), and high-flow nasal cannula (0.05%). Patients with PRF left the operating room while receiving MV more often than patients without PRF (49.8% vs 4.5%) and while receiving room air less often (14.2% vs 46.3%; *P* < .001). Patients with PRF underwent a median of 164 h of postoperative MV (Table 2). Nearly one-half of patients with PRF continued to receive MV for > 48 h immediately after surgery, whereas 52% were reintubated and returned to MV for > 48 h. The median time to reintubation for patients with PRF was 51.4 h.

Ninety-nine percent of patients with PRF were admitted to an ICU from the operating room, compared with only 17% of patients without PRF (*P* < .001). Patients with PRF underwent longer hospital and ICU lengths of stay (Table 3). Twenty-four percent of patients with PRF died in the hospital, compared to ≤ 1% of patients without PRF. Of the 171 patients with PRF who survived to discharge, 95 patients (42%) were discharged to another facility (eg, skilled nursing, long-term acute care), rather than home.

### Predictive Model Performance

The LASSO procedure retained 18 predictors in the logistic regression (Table 4). Duration of anesthesia (hours), net fluid balance at operating room departure (liters), operations on the cardiovascular system, Medicare (as the primary payer), and American Society of Anesthesiologists class of ≥ III were selected as predictors in all bootstrap samples and increased the odds of PRF. Other predictors included operations on the cardiovascular, nervous, digestive, urinary, or musculoskeletal system; surgical specialty orthopedic (nonspine); Medicaid (as the primary payer); race unknown; BMI of 30 to 34.9 kg/m^2^; median FIO_2_, end-tidal CO_2_ (EtCO_2_), heart rate, and tidal volume; and intraoperative vasopressor medications. All predictors except race unknown and EtCO_2_ were retained in ≥ 80% of bootstrap samples (Table 4).

**Figure 3 –:**
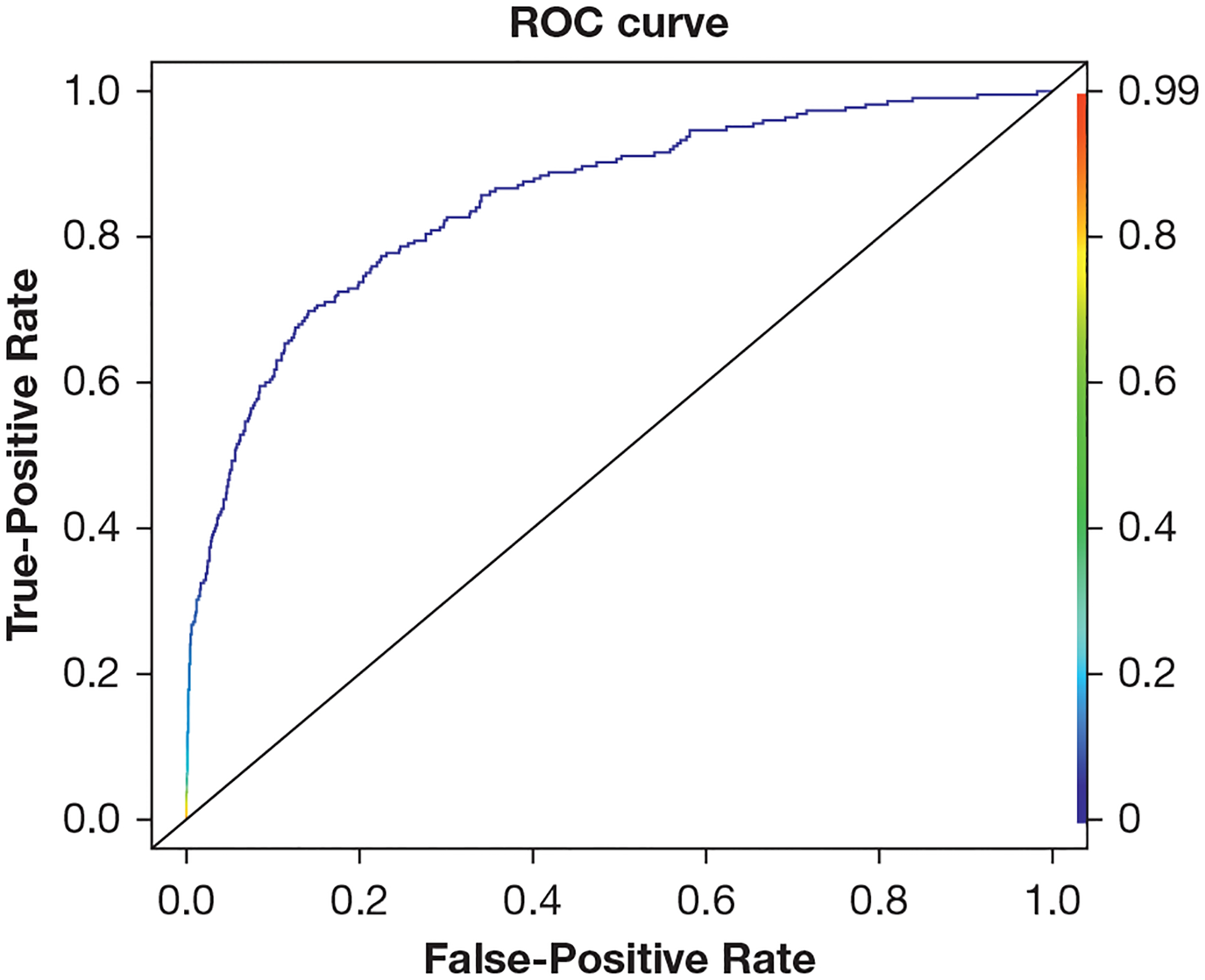
ROC curve for fitted least absolute shrinkage and selection operator logistic regression predicting postoperative respiratory failure. This model achieved an observed area under the ROC curve (AUC) of 0.851 (95% CI, 0.824–0.878) and an optimism-corrected AUC of 0.835 (95% CI, 0.808–0.862). AUC = area under the operating curve; ROC = receiver operating characteristic.

**Figure 4 –:**
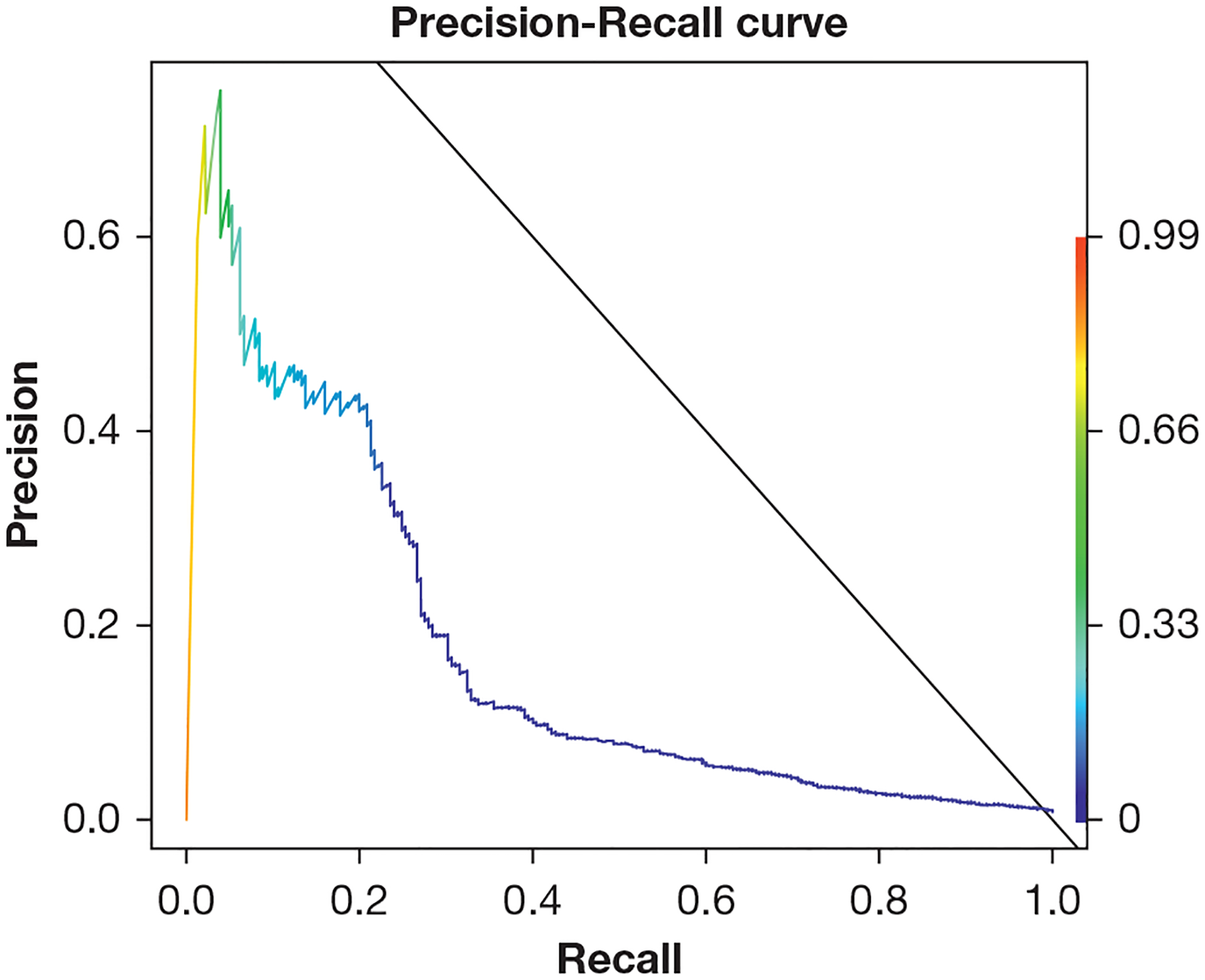
Precision-recall curve for fitted least absolute shrinkage and selection operator logistic regression predicting postoperative respiratory failure. This model achieved an observed area under the precision-recall curve of 0.174 (95% CI, 0.123–0.221) with an optimism-corrected value of 0.156 (95% CI, 0.105–0.203).

**Figure 5 –:**
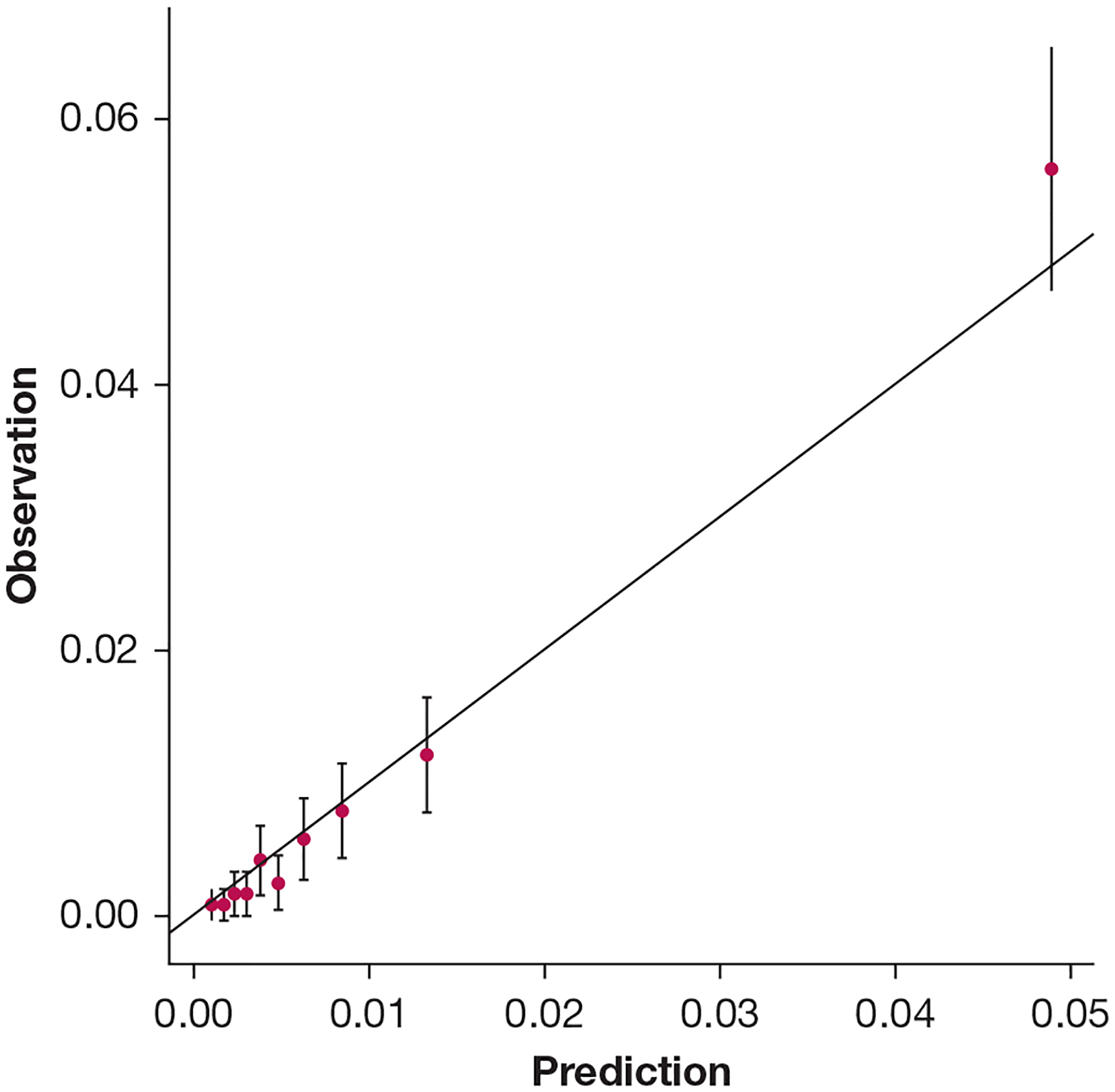
Calibration plot for the least absolute shrinkage and selection operator logistic regression model predicting postoperative respiratory failure. To create this plot, predicted probabilities were binned into 10 equally sized groups. The mean predicted probability and 95% CI were calculated for each bin and were plotted against the observed proportion of events in each bin. Because of the very low prevalence of events, the mean predicted probability remains small (approximately 5%), even for the bin containing the largest predicted probabilities. The mean predicted probabilities are close to the 45° line, reflecting good agreement between predicted probabilities and observed probabilities, and hence good calibration.

This model achieved an observed AUC of 0.851 (95% CI, 0.824–0.878) and an optimism-corrected AUC of 0.835 (95% CI, 0.808–0.862) (Fig 3). The observed AUPRC was 0.174 (95% CI, 0.123–0.221) with an optimism-corrected value of 0.156 (95% CI, 0.105–0.203) (Fig 4). The calibration curve indicates that the predicted probabilities are a strong match for the actual outcomes (Fig 5).

We used Youden’s index^27^ to identify a potential threshold for discriminating patients with PRF from patients without PRF. A predicted probability of PRF of 1.315% maximized Youden’s index, achieving an optimism-corrected sensitivity of 0.647 (95% CI, 0.593–0.713) and specificity of 0.858 (95% CI, 0.851–0.86) (Table 5). Other performance metrics (positive predictive value, negative predictive value, Brier score) are provided in Table 5. The confusion matrix shows 3,372 of 23,774 as false-positive findings and 69 of 225 as false-negative findings (Table 6).

### Sensitivity and Robustness Analyses

In the secondary analyses (e-Appendix 2), the predictors retained in the LASSO logistic regression and their coefficients like were the primary model (e-Table 2). Performance metrics of models developed with the training set and applied to the holdout test sets were slightly worse than the optimism-corrected metrics for the primary model. The AUC declined from 0.835 to between 0.763 and 0.786 in the supplementary analyses, whereas the AUPRC values increased from 0.156 for the primary model to 0.172 in the comparable secondary analysis (e-Table 3, approach 1). We also performed sensitivity analysis to determine the effect of including Elixhauser comorbidity count and score on model performance (e-Appendix 3; e-Table 5, e-Figure 1, e-Figure 2). This resulted in a 13-variable predictive model with a negligible increase in optimism-corrected AUC from 0.835 to 0.84 and an AUPRC from 0.156 to 0.162 (e-Table 4).

## Discussion

We developed a prediction model for PRF that used readily available patient preoperative and intraoperative data from 23,999 consecutive adult elective surgeries using an automated SQL ETL process. Our model includes 18 variables; duration of anesthesia, net fluid balance at operating room departure, operations on the cardiovascular system, Medicare coverage, and American Society of Anesthesiologists class ≥ III were selected as predictors in all bootstrap samples. Other predictors included operations on the cardiovascular, nervous, digestive, urinary, or musculoskeletal system; surgical specialty orthopedic (nonspine); Medicaid coverage; race unknown; BMI of 30 to 34.9 kg/m^2^; median FIO_2_, EtCO_2_, heart rate, and tidal volume; and intraoperative vasopressor medications. The model showed good discrimination and calibration. Secondary analyses validated our primary findings.

This study extends prior work in several important ways. In contrast to our previous PRF research that used manual chart abstraction,^28–30^ our current study developed and validated an automated ETL process to enable efficient, standardized acquisition of real-world data from the EHR. The potentially extensible nature of SQL ETL processes should allow adaptation of our methods to the EHRs of other research sites, thereby enabling data acquisition and large-scale research into rare events like PRF that would not be feasible if data collection were restricted to manual chart review. Although our prior work focused on developing an explanatory model, our current study aimed to develop a model optimized for prediction that eventually might be incorporated into clinical decision support (CDS)-aided clinical workflows. Our work is distinct from the work of others in that we excluded emergent surgical procedures and preoperative laboratory findings and focused exclusively on elective surgical procedures. We also narrowed our outcome of interest to PRF, rather than the broad continuum of all postoperative pulmonary complications.

In this predictive model, we aimed to estimate accurately the probability that PRF would develop based on preoperative and intraoperative factors. Other published predictive models (eg, Assess Respiratory Risk in Surgical Patients in Catalonia [ARISCAT],^18^ Prospective Evaluation of a Risk Score for Postoperative Pulmonary Complications in Europe [PERISCOPE],^19^ and Local Assessment of Ventilatory Management During General Anesthesia for Surgery [LAS VEGAS]^20^) focused on all postoperative pulmonary complications, ranging from atelectasis to respiratory failure, which occurred in 5% to 11% of patients. These models also included emergency surgeries. Despite the good discrimination of all three models, the focus on all postoperative pulmonary complications and the inclusion of emergency surgeries makes extrapolation to elective surgery populations challenging and external validation of the models in the patient population impossible. Importantly, the ARISCAT and PERISCOPE studies did not include intraoperative fluid, medications, or MV parameters in their predictive models. The LAS VEGAS study evaluated intraoperative predictors, but the inclusion of emergency surgeries precludes direct comparison with our model. The more recent Respiratory Support, Prolonged Intubation, or Reintubation. Accuracy (RESPIRE)^17^ single-site predictive model for PRF was EHR based and had good accuracy; however, in addition to using a consensus definition for PRF that differed from ours, it included outpatient, same-day, and emergency surgeries and did not include intraoperative treatment factors, although surgical site was included.

To create a targeted and readily interpretable model for CDS, we chose a fundamentally different approach by considering both pre-existing patient comorbidities and intraoperative treatment. Our goal was to consider the effect of a patient’s pre-existing risk factors, intraoperative care and physiologic parameters, and status on exiting the operating room to determine risk and to assist in postoperative level of care and treatment decisions. This approach is congruent with the theory of cascade iatrogenesis,^31,32^ in which adverse events may occur if trigger events are not recognized and addressed. An example of cascade iatrogenesis is intraoperative fluid overload in a patient with pre-existing heart failure, leading to pulmonary edema, respiratory failure, and invasive MV. We also chose a different statistical approach than others, logistic regression, because we sought to develop a model that was readily interpretable by clinicians and that could be developed into a risk score-based, real-time CDS tool.

Possible clinical applications of our model include identification of at-risk patients who could benefit from postoperative admission or upgrade to the ICU; implementation and monitoring of adherence to the daily Assess, Prevent, and Manage Pain, Both Spontaneous Awakening Trials and Spontaneous Breathing Trials, Choice of Analgesia and Sedation, Delirium: Assess, Prevent, and Manage, Early Mobility and Exercise, and Family Engagement and Empowerment bundle^33^; and the postoperative application of procedure-specific, evidence-based enhanced recovery after surgery^34^ protocols. For example, although enhanced recovery after surgery implementation has been shown to improve outcomes in almost all major surgical specialties,^34^ as a multidisciplinary and multimodal approach, it can be resource intensive, thus limiting its widespread use. Application of well-calibrated PRF prediction models may allow patient-level risk stratification and subsequent ICU admission; Assess, Prevent, and Manage Pain, Both Spontaneous Awakening Trials and Spontaneous Breathing Trials, Choice of Analgesia and Sedation, Delirium: Assess, Prevent, and Manage, Early Mobility and Exercise, and Family Engagement and Empowerment bundle implementation; and enhanced recovery after surgery application for only those patients identified as at risk, simultaneously optimizing patient outcomes and the efficiency of care delivery by avoiding underuse or overuse of critical care resources.^35^ Early identification of patients at risk of PRF, creation of supportive infrastructure, and implementation of prevention strategies helped one health system reduce PRF by 35%.^36^

Strengths of our study include our easily interpretable statistical approach, use of a large and diverse patient population, and restriction to elective surgeries and the outcome of PRF to reduce heterogeneity. Our development of an SQL ETL data extraction method enabled us to analyze all 23,999 consecutive elective surgical encounters over an 8-year period. This approach could improve the ability to build scale in studies of PRF and to support implementation and validation of predictive models across health systems. Our focus on a more narrowly defined population and single serious adverse event should enable future researchers both to refine predictive models and to test the effects of incorporating model outputs into CDS-enabled clinical workflows designed to prevent adverse outcomes such as PRF in at-risk patients.^37,38^

Limitations of our current study include the single-center proof-of-concept design and a relatively small number of patients with PRF, which we addressed through optimism-corrected analyses. With our SQL ETL, we were limited to analyses of data found in discrete fields, rather than free-text notes. This constrained our definition of the primary outcome to MV after surgery of > 48 h without further qualification of the reason for prolonged MV. Thus, it is possible this cohort of 225 patients with PRF includes patients who required prolonged MV for airway protection, not respiratory failure. In our prior work, 4.3% of patients flagged for PRF had airway compromise, not respiratory failure.^28^ We also acknowledge that not all cases of PRF can be prevented. Patients at risk may still opt to undergo an elective surgical intervention to address quality-of-life issues such as chronic pain or reduced life expectancy (eg, laminectomy, lung resection). Furthermore, our ETL procedure was developed in a standard EHR deployment from a single vendor, and it is possible that extension of our methods to a nonstandard Epic implementation or another EHR vendor’s data model would require cost-prohibitive adaptation of our methods. Finally, the model was developed using data from one hospital, and external validation in other cohorts is needed to confirm its performance.

Feasible multicenter analysis is key to the study of rare adverse events such as PRF. We have described a method using an SQL ETL that could be deployed at other centers effectively to automate the abstraction of tens of thousands of charts, work that would not be feasible through manual chart abstraction. The ability to predict patients at risk of PRF reliably using readily available patient preoperative and intraoperative variables is valuable for clinicians and may afford individualized, optimized postoperative planning. Future research is needed to validate our findings in other centers, to conduct clustered machine learning to identify subgroups (eg, low, moderate, and high risk), and to develop, test, and operationalize a risk score for real-time use by clinicians.

In conclusion, we developed a prediction model for PRF based on readily available patient, preoperative, and intraoperative data using an automated procedure to extract large volumes of data from the EHR. If validated in other centers, our model may represent an intuitive and practical tool for prediction of PRF. With improved prediction, clinician scientists can understand PRF better, can begin to classify phenotypes, and can discern if heterogeneity of treatment effect exists. This eventually might lead to improved care and outcomes for PRF, which is associated with high morbidity and mortality.

## Supplementary Material

1

## Figures and Tables

**TABLE 1]:** Patient Characteristics: Patients Who Demonstrated PRF Compared With Patients Who Did Not Demonstrate PRF

Variable	Total Sample (N = 23,999)	No PRF (n = 23,774)	All PRF (n = 225)	*P* Value^a^
Patient demographics				
Age, y	61 (49–70)	61 (49–70)	66 (56–73)	< .001
Sex, male	11,237 (46.8)	11,111 (46.7)	126 (56.0)	.006
Race				.310
White	17,123 (71.3)	16,973 (71.4)	150 (66.7)	
Black (Black and African American)	1,396 (5.8)	1,382 (5.8)	14 (6.2)	
Asian (Asian, Native Hawaiian or Pacific Islander, American Indian)	1,379 (5.7)	1,361 (5.7)	18 (8.0)	
Other (other, multiracial)	3,733 (15.6)	3,696 (15.5)	37 (16.4)	
Unknown (unable, unavailable, unknown, declined)	368 (1.5)	362 (1.5)	6 (2.7)	
Ethnicity				.319
Non-Hispanic	20,559 (85.7)	20,373 (85.7)	186 (82.7)	
Hispanic	2,799 (11.7)	2,769 (11.6)	30 (13.3)	
Not reported (unknown)	641 (2.7)	632 (2.7)	9 (4.0)	
Primary payer category				< .001
Medicare	10,844 (45.2)	10,701 (45.0)	143 (63.6)	
Medicaid	3,633 (15.1)	3,589 (15.1)	44 (19.6)	
Commercial insurance	9,416 (39.2)	9,379 (39.5)	37 (16.4)	
Unavailable/unknown	106 (0.4)	105 (0.4)	1 (0.4)	
Patient comorbidities				
BMI, kg/m^2^				< .001
< 18.5 (underweight)	534 (2.3)	519 (2.2)	15 (6.7)	
18.5–24.9 (normal)	6,197 (26.2)	6,126 (26.1)	69 (30.8)	
25–29.9 (overweight)	7,409 (31.3)	7,338 (31.3)	71 (31.7)	
30–34.9 (obese 1)	5,007 (21.2)	4,973 (21.2)	34 (15.2)	
35–39.9 (obese 2)	2,530 (10.7)	2,512 (10.7)	18 (8.0)	
≥ 40 (morbidly obese)	1,986 (8.4)	1,969 (8.4)	17 (7.6)	
ASA class, ≥ III	15,957 (66.7)	15,746 (66.4)	211 (93.8)	< .001
Elixhauser comorbidity score^b^	1 (0–8)	1 (0–8)	11 (4–17)	< .001
Elixhauser comorbidity count^b^	2 (1–4)	2 (1–4)	4 (3–7)	< .001

Data are presented as No. (%) or median (interquartile range), unless otherwise indicated. ASA = American Society of Anesthesiologists; PRF = postoperative respiratory failure.

a Wilcoxon rank-sum test for continuous variables and Pearson χ^2^ test for categorical variables.

b Comorbid conditions included in the Elixhauser measures: congestive heart failure, cardiac arrhythmias, valvular disease, pulmonary circulatory disorders, peripheral vascular disorders, hypertension (uncomplicated), hypertension (complicated), paralysis, other neurologic disorders, chronic pulmonary disease, diabetes (uncomplicated), diabetes (complicated), hypothyroidism, renal failure, liver disease, peptic ulcer disease excluding bleeding, AIDS or HIV, lymphoma, metastatic cancer, solid tumor without metastasis, rheumatoid arthritis or collagen vascular diseases, coagulopathy, obesity, weight loss, fluid and electrolyte disorders, blood loss anemia, deficiency anemia, alcohol abuse, drug abuse, psychoses, and depression.^25^ Elixhauser comorbidity score was calculated by assigning weights to each comorbidity based on van Walraven et al.^26^

**TABLE 2]:** Perioperative Characteristics: Patients Who Demonstrated PRF Compared With Patients Who Did Not Demonstrate PRF

Variable	Total Sample (N = 23,999)	No PRF (n = 23,774)	All PRF (n = 225)	*P* Value^a^
Anesthesia procedure				
Anesthesia duration, h	4.6 (3.3–6.4)	4.6 (3.3–6.3)	8.8 (5.8–11.4)	< .001
Surgical specialty				< .001
General	4,706 (19.6)	4,678 (19.7)	28 (12.4)	
Cardiovascular	2,898 (12.1)	2,807 (11.8)	91 (40.4)	
Neurosurgery, including spine	3,520 (14.7)	3,487 (14.7)	33 (14.7)	
Oncology	2,200 (9.2)	2,179 (9.2)	21 (9.3)	
Orthopedic, nonspine	5,441 (22.7)	5,431 (22.8)	10 (4.4)	
Urology, gynecology	3,336 (13.9)	3,311 (13.9)	25 (11.1)	
Head, eyes, ears, nose, throat	1,880 (7.8)	1,863 (7.8)	17 (7.6)	
Other	18 (0.1)	18 (0.1)	0 (0)	
Operations by ICD coding system				< .001
Cardiovascular	1,554 (6.4)	1,465 (6.2)	79 (35.1)	
Digestive	4,165 (17.4)	4,118 (17.3)	47 (20.9)	
Ear	105 (0.4)	105 (0.4)	0 (0)	
Endocrine	678 (2.8)	676 (2.8)	2 (0.9)	
Eye	29 (0.1)	29 (0.1)	0 (0)	
Female genital organs	1,494 (6.2)	1,489 (6.3)	5 (2.2)	
Hemic and lymphatic	675 (2.8)	671 (2.8)	4 (1.8)	
Integumentary	1,164 (4.9)	1,160 (4.9)	4 (1.8)	
Male genital organs	428 (1.8)	428 (1.8)	0 (0)	
Musculoskeletal	7,087 (29.5)	7,062 (29.7)	25 (11.1)	
Nervous	1,846 (7.7)	1,821 (7.7)	25 (11.1)	
Nose, mouth, and pharynx	499 (2.1)	495 (2.1)	4 (1.8)	
Obstetric	51 (0.2)	51 (0.2)	0 (0)	
Respiratory	1,022 (4.3)	1,012 (4.3)	10 (4.4)	
Urinary	1,882 (7.8)	1,862 (7.8)	20 (8.9)	
Miscellaneous diagnostic and therapeutic procedures	8 (< 0.1)	8 (< 0.1)	0 (0)	
Unable to map to ICD clinical classification system because of annual updates to technical specification and multiyear dataset	1,322 (5.5)	1,322 (5.6)	0 (0)	
Surgical duration, h	3.3 (2.1–4.8)	3.2 (2.1–4.8)	6.7 (4.1–9.4)	< .001
Intraoperative ventilator management				
Tidal volume, mL	500 (434–550)	500 (435–550)	475 (421–536)	.004
Positive end-expiratory pressure, cm H_2_O	5 (5–5)	5 (5–5)	5 (5–5)	.004
Positive end-expiratory pressure > 5 cm H_2_O	4,212 (18.1)	4,158 (18.0)	54 (24.3)	.05
Peak inspiratory pressure, cm H_2_O	19 (16–23)	19 (16–23)	19 (17–23)	.157
Plateau pressure, cm H_2_O	18 (16–20)	18 (16–19)	21 (16–28)	.359
No. of patients	32	26	6	
Respiratory rate	12 (10–12)	12 (10–12)	12 (10–13)	.004
Fio_2_	0.56 (0.49–0.61)	0.56 (0.49–0.61)	0.60 (0.53–0.84)	< .001
Oxygen saturation, %	99 (98–100)	99 (98–100)	100 (99–100)	< .001
EtCO_2_, mm Hg	35 (33–37)	35 (33–37)	34 (32–36)	< .001
Intraoperative fluid management				
Net fluid in operating room, L	1.3 (0.8–2.0)	1.3 (0.8–2.0)	2.3 (1.1–5.5)	< .001
Net fluid in first 24 h after surgery, L	0.28 (-0.6 to 1.1)	0.27 (-0.6 to 1.1)	1.17 (0.1–2.8)	< .001
Intraoperative medication management				
Morphine equivalent units, total mg	90 (71–127)	90 (71–126)	147 (82–225)	< .001
Vasopressor medications administered in the operating room (includes bolus dose and continuous infusions), yes/no	15,112 (63.0)	14,912 (62.7)	200 (88.9)	< .001
Vasopressor medications as a continuous, titrated infusion, yes/no	4,721 (19.7)	4,594 (19.3)	127 (56.4)	< .001
Vasopressor medications as a continuous, titrated infusion, count	1 (1–1)	1 (1–1)	1 (1–3)	< .001
No. (%) of patients	4,721 (19.7)	4,594 (19.3)	127 (56.4)	
Phenylephrine infusion	3,778 (15.7)	3,718 (15.6)	60 (26.7)	< .001
Norepinephrine infusion	902 (3.8)	834 (3.5)	68 (30.2)	< .001
Vasopressin infusion	249 (1.0)	208 (0.9)	41 (18.2)	< .001
Dopamine infusion	87 (0.4)	75 (0.3)	12 (5.3)	< .001
Dobutamine infusion	20 (0.1)	18 (0.1)	2 (0.9)	< .001
Milrinone infusion	81 (0.3)	60 (0.3)	21 (9.3)	< .001
Intraoperative vital signs				
Mean arterial pressure, mm Hg	74 (68–80)	74 (68–80)	73 (68–79)	.368
Heart rate, beats/min	69 (63–77)	69 (63–77)	73 (65–80)	< .001
Mechanical ventilation duration				
First continuous phase after operating room, h	0 (0–0)	0 (0–0)	88.4 (51–177.7)	< .001
Longest continuous phase after operating room, h	0 (0–0)	0 (0–0)	138.6 (78.6–278.0)	< .001
Total duration of all continuous phases after operating room, h	0 (0–0)	0 (0–0)	163.8 (86.5–401.7)	< .001
Reintubation after operating room				
Reintubated after operating room	380 (1.6)	263 (1.1)	117 (52.0)	< .001
Time from airway removal to reintubation after operating room, h	0 (0–0)	0 (0–0)	51.4 (0–135.1)	< .001

Data are presented as No. (%) or median (interquartile range), unless otherwise indicated. EtCO_2_ = end-tidal carbon dioxide; ICD = International Classification of Diseases; PRF postoperative respiratory failure.

a Wilcoxon rank-sum test for continuous variables and Pearson’s χ^2^ test for categorical variables.

**TABLE 3]:** Outcomes for Patients Who Demonstrated PRF Compared With Patients Who Did Not Demonstrate PRF

Variable	Total Sample (N = 23,999)	No PRF (n = 23,774)	All PRF (n = 225)	*P* Value^a^
Hospital length of stay, d	3.3 (1.9–5.3)	3.3 (1.8–5.3)	21.5 (13.4–41.2)	< .001
ICU stay, yes/no	4,136 (17.2)	3,914 (16.5)	222 (98.7)	< .001
ICU length of stay (for those who had an ICU stay), d	2.01 (0.98–3.97)	1.93 (0.96–3.75)	13.6 (8.1–27.1)	< .001
Total No. of trips to operating room, mean (SD)	1.02 (0.2)	1.01 (0.1)	1.29 (0.7)	< .001
> 1 total trip to the operating room	323 (1.3)	279 (1.2)	44 (19.6)	< .001
Discharge disposition				< .001
Home	21,693 (90.4)	21,617 (90.9)	76 (33.8)	
Died	100 (0.4)	46 (0.2)	54 (24.0)	
Discharge to other facility (SNF, LTAC, other acute care)	2,206 (9.2)	2,111 (8.9)	95 (42.2)	

Data are presented as No. (%) or median (interquartile range), unless otherwise indicated. LTAC = long-term acute care; PRF = postoperative respiratory failure; SNF = skilled nursing facility.

a Wilcoxon rank-sum test for continuous variables and Pearson χ^2^ test for categorical variables.

**TABLE 4]:** Variables Retained by the LASSO Procedures in the Logistic Regression for Predicting Occurrence of PRF

Predictor Variable	Coefficient	Probability Selected, %^a^
Intercept	−7.4426	100
Anesthesia duration, /h	0.1921	100
Net fluid at end of the operation, /L	0.1681	100
Operations on the cardiovascular system	0.9246	100
Medicare (as the primary payer)	0.4672	100
ASA class ≥ III	0.5663	100
Fio_2_, median	0.0086	98
Operations on the nervous system	0.5520	96.4
Vasopressor medication in the operating room	0.3000	95.6
Heart rate, median	0.0088	94.8
Tidal volume, median	−0.0011	92.4
Operations on the musculoskeletal system	−0.3957	91.2
Operations on the digestive system	0.1136	88.8
Surgical specialty orthopedic (nonspine)	−0.2202	82.8
Medical (Medicaid) (as the primary payer)	0.1937	82.4
Operations on the urinary system	0.1706	81.2
BMI 30–34.9 kg/m^2^ (obese 1)	−0.0745	80.4
End-tidal CO_2_, median	−0.0135	75.6
Race unknown (unable to respond, unavailable or unknown, declined to state)	0.2557	62.8

ASA = American Society of Anesthesiologists; LASSO = least absolute shrinkage and selection operator; PRF = postoperative respiratory failure;

a Probability selected is the percentage of bootstrap samples in which the variable was retained.

**TABLE 5]:** Observed and Optimism-Corrected Performance Metrics for the LASSO Logistic Regression Predicting PRF

Variable	Sensitivity	Specificity	PPV	NPV	AUC	AUPRC	Brier Score
Observed performance (original data performance)^a,b^	0.693 (0.638–0.758)	0.858 (0.851–0.86)	0.042 (0.037–0.05)	0.997 (0.996–0.997)	0.851 (0.824–0.878)	0.174 (0.123–0.221)	0.008
Optimism-corrected performance	0.647 (0.593–0.713)	0.858 (0.851–0.86)	0.042 (0.034–0.048)	0.996 (0.995–0.997)	0.835 (0.808–0.862)	0.156 (0.105–0.203)	0.009
Estimated optimism	0.045	0	0.003	0.001	0.016	0.018	0

Data are presented as value (95% CI). AUC = area under the receiver operating characteristic curve; AUPRC = area under the precision-recall curve; LASSO = least absolute shrinkage and selection operator; NPV = negative predictive value; PPV = positive predictive value; PRF = postoperative respiratory failure.

a Values for sensitivity, specificity, PPV, and NPV are based on a threshold of 1.315%.

b Performance table of original data with probability criterion selected by Youden′s index, optimism-corrected performance, and estimated stable optimism

**TABLE 6]:** Confusion Matrix of Predicted Patients With PRF and Patients Without PRF^a^

Variable	Patients Without PRF	Patients With PRF
Predicted no PRF	20,402	69
Predicted PRF	3,372	156

Data are presented as No. PRF = postoperative respiratory failure.

a Using 1.315% as classification threshold.

## Data Availability

The data sets used or analyzed during the current study are available from the corresponding author on reasonable request.

## Take-home Points

### Study Question:

In this study, we sought to determine if a predictive model, using readily available patient and intraoperative factors, could identify patients at high risk of postoperative respiratory failure accurately.

### Results:

We developed an 18-variable predictive model for PRF that included operations on the cardiovascular, nervous, digestive, urinary, or musculoskeletal system; surgical specialty orthopedic (nonspine); Medicare or Medicaid (as the primary payer); race unknown; American Society of Anesthesiologists class ≥ III; BMI of 30 to 34.9 kg/m^2^; anesthesia duration (per hour); net fluid at end of the operation (per liter); median intraoperative FIO_2_, end-tidal carbon dioxide, heart rate, and tidal volume; and intraoperative vasoactive medications.

### Interpretation:

A predictive model for postoperative respiratory failure, based on readily available patient and intraoperative variables, achieved an optimism-corrected area under the receiver operating characteristic curve of 0.835 (95% CI, 0.808–0.862) and an area under the precision-recall curve of 0.156 (95% CI, 0.105–0.203).

## ABBREVIATIONS:

AUC: area under the receiver operating characteristic curve
AUPRC: area under the precision-recall curve
CDS: clinical decision support
EHR: electronic health record
EtCO_2_: end-tidal CO_2_
ETL: extract; transform = and load
LASSO: least absolute shrinkage and selection operator
MV: mechanical ventilation
PRF: postoperative respiratory failure
SQL: structured query language

## References

[1] Gupta H, Gupta PK, Fang X, Development and validation of a risk calculator predicting postoperative respiratory failure. Chest. 2011;140(5):1207–1215. 21757571 10.1378/chest.11-0466

[2] Arozullah AM, Daley J, Henderson WG, Khuri SF. Multifactorial risk index for predicting postoperative respiratory failure in men after major noncardiac surgery. The National Veterans Administration Surgical Quality Improvement Program. Ann Surg. 2000;232(2):242–253. 10903604 10.1097/00000658-200008000-00015 PMC1421137

[3] Canet J, Sabate S, Mazo V, Development and validation of a score to predict postoperative respiratory failure in a multicentre European cohort: a prospective, observational study. Eur J Anaesthesiol. 2015;32(7):458–470. 26020123 10.1097/EJA.0000000000000223

[4] Kor DJ, Lingineni RK, Gajic O, Predicting risk of postoperative lung injury in high-risk surgical patients: a multicenter cohort study. Anesthesiology. 2014;120(5):1168–1181. 24755786 10.1097/ALN.0000000000000216 PMC3999474

[5] Zhan C, Miller MR. Excess length of stay, charges, and mortality attributable to medical injuries during hospitalization. JAMA. 2003;290(14):1868–1874. 14532315 10.1001/jama.290.14.1868

[6] Encinosa WE, Hellinger FJ. What happens after a patient safety event? Medical expenditures and outcomes in Medicare. In: Henriksen K, Battles JB, Marks ES, Lewin DI, eds. Advances in Patient Safety: From Research to Implementation (Volume 1: Research Findings). Agency for Healthcare Research and Quality (US); 2005:423–436. 21249795

[7] Encinosa WE, Hellinger FJ. The impact of medical errors on ninety-day costs and outcomes: an examination of surgical patients. Health Serv Res. 2008;43(6):2067–2085. 18662169 10.1111/j.1475-6773.2008.00882.x PMC2613997

[8] Carey K, Stefos T, Shibei Z, Borzecki AM, Rosen AK. Excess costs attributable to postoperative complications. Med Care Res Rev. 2011;68(4):490–503. 21536599 10.1177/1077558710396378

[9] Kor DJ, Warner DO, Alsara A, Derivation and diagnostic accuracy of the surgical lung injury prediction model. Anesthesiology. 2011;115(1):117–128. 21694510 10.1097/ALN.0b013e31821b5839 PMC3986041

[10] Johnson RG, Arozullah AM, Neumayer L, Henderson WG, Hosokawa P, Khuri SF. Multivariable predictors of postoperative respiratory failure after general and vascular surgery: results from the patient safety in surgery study. J Am Coll Surg. 2007;204(6):1188–1198. 17544077 10.1016/j.jamcollsurg.2007.02.070

[11] Johnson AP, Altmark RE, Weinstein MS, Pitt HA, Yeo CJ, Cowan SW. Predicting the risk of postoperative respiratory failure in elective abdominal and vascular operations using the National Surgical Quality Improvement Program (NSQIP) participant use data file. Ann Surg. 2016;266(6):968–974. 10.1097/SLA.0000000000001989 27607099

[12] Cote DJ, Karhade AV, Burke WT, Larsen AM, Smith TR. Risk factors for post-operative respiratory failure among 94,621 neurosurgical patients from 2006 to 2013: a NSQIP analysis. Acta Neurochir (Wien). 2016;158(9):1639–1645. 27339268 10.1007/s00701-016-2871-8

[13] Filsoufi F, Rahmanian PB, Castillo JG, Chikwe J, Adams DH. Predictors and early and late outcomes of respiratory failure in contemporary cardiac surgery. Chest. 2008;133(3):713–721. 18263692 10.1378/chest.07-1028

[14] Leisman DE, Harhay MO, Lederer DJ, Development and reporting of prediction models: guidance for authors from editors of respiratory, sleep, and critical care journals. Crit Care Med. 2020;48(5):623–633. 32141923 10.1097/CCM.0000000000004246 PMC7161722

[15] von Elm E, Altman DG, Egger M, Pocock SJ, Gøtzsche PC, Vandenbroucke JP. The Strengthening the Reporting of Observational Studies in Epidemiology (STROBE) statement: guidelines for reporting observational studies. Ann Internal Med. 2007;147(8):573–577. 17938396 10.7326/0003-4819-147-8-200710160-00010

[16] Collins GS, Reitsma JB, Altman DG, Moons KG. Transparent reporting of a multivariable prediction model for individual prognosis or diagnosis (TRIPOD): the TRIPOD statement. BMC Med. 2015;13:1–10. 25563062 10.1186/s12916-014-0241-z PMC4284921

[17] Kiyatkin ME, Aasman B, Fazzari MJ, Development of an automated, general-purpose prediction tool for postoperative respiratory failure using machine learning: a retrospective cohort study. J Clin Anesth. 2023;Nov(90):1–10. 10.1016/j.jclinane.2023.111194 PMC10529165 37422982

[18] Canet J, Gallart L, Gomar C, Prediction of postoperative pulmonary complications in a population-based surgical cohort. Anesthesiology. 2010;113(6):1338–1350. 21045639 10.1097/ALN.0b013e3181fc6e0a

[19] Mazo V, Sabaté S, Canet J, Prospective external validation of a predictive score for postoperative pulmonary complications. Anesthesiology. 2014;121(2):219–231. 24901240 10.1097/ALN.0000000000000334

[20] Neto AS, da Costa LGV, Hemmes SNT, The LAS VEGAS risk score for prediction of postoperative pulmonary complications: an observational study. Eur J Anaesthesiol. 2018;35(9):691–701. 29916860 10.1097/EJA.0000000000000845 PMC7450515

[21] Tibshirani R Regression shrinkage and selection via the LASSO. J R Stat Soc Series B Methodol. 1996;58(1):267–288.

[22] Krstajic D, Buturovic LJ, Leahy DE, Thomas S. Cross-validation pitfalls when selecting and assessing regression and classification models. J Cheminform. 2014;6(1):1–15. 24678909 10.1186/1758-2946-6-10 PMC3994246

[23] Steyerberg EW. Clinical Prediction Models: A Practical Approach to Development, Validation and Updating. Springer Science+Business Media, LLC; 2019:1–497.

[24] Chatterjee A, Lahiri SN. Bootstrapping lasso estimators. J Am Stat Assoc. 2011;106(494):608–625.

[25] Quan H, Sundararajan V, Halfon P, Coding algorithms for defining comorbidities in ICD-9-CM and ICD-10 administrative data. Med Care. 2005;43(11):1130–1139. 16224307 10.1097/01.mlr.0000182534.19832.83

[26] van Walraven C, Austin PC, Jennings A, Quan H, Forster AJ. A modification of the Elixhauser comorbidity measures into a point system for hospital death using administrative data. Med Care. 2009;47(6):626–633. 19433995 10.1097/MLR.0b013e31819432e5

[27] Steyerberg EW, Vickers AJ, Cook NR, Assessing the performance of prediction models: a framework for traditional and novel measures. Epidemiology. 2010;21(1):128–138. 20010215 10.1097/EDE.0b013e3181c30fb2 PMC3575184

[28] Stocking JC, Utter GH, Drake C, Postoperative respiratory failure: an update on the validity of the Agency for Healthcare Research and Quality Patient Safety Indicator 11 in an era of clinical documentation improvement programs. Am J Surg. 2020;220(1):222–228. 31757440 10.1016/j.amjsurg.2019.11.019 PMC10091853

[29] Stocking JC, Drake C, Aldrich JM, Risk factors associated with early postoperative respiratory failure: a matched case-control study. J Surg Res. 2021;261:310–319. 33485087 10.1016/j.jss.2020.12.043 PMC10062707

[30] Stocking JC, Drake C, Aldrich JM, Outcomes and risk factors for delayed-onset postoperative respiratory failure: a multi-center case-control study by the University of California Critical Care Research Collaborative (UC3RC). BMC Anesthesiol. 2022;22(1):1–12. 35568812 10.1186/s12871-022-01681-x PMC9107656

[31] Thornlow DK, Oddone E, Anderson R. Cascade iatrogenesis: a case-control study to detect postoperative respiratory failure in hospitalized older adults. Res Gerontol Nurs. 2014;7(2):66–77. 24297156 10.3928/19404921-20131126-01

[32] Thornlow DK, Anderson R, Oddone E. Cascade iatrogenesis: factors leading to the development of adverse events in hospitalized older adults. Int J Nurs Stud. 2009;46(11):1528–1535. 19643409 10.1016/j.ijnurstu.2009.06.015

[33] Ely EW. The ABCDEF bundle: science and philosophy of how ICU liberation serves patients and families. Crit Care Med. 2017;45(2):321–330. 28098628 10.1097/CCM.0000000000002175 PMC5830123

[34] Ljungqvist O, Scott M, Fearon KC. Enhanced recovery after surgery: a review. JAMA Surg. 2017;152(3):292–298. 28097305 10.1001/jamasurg.2016.4952

[35] Sobol JB, Wunsch H. Triage of high-risk surgical patients for intensive care. Crit Care. 2011;15(2):438–446. 21457500 10.1186/cc9999 PMC3219413

[36] Hill J, Asher D, Zanath E, A hospital system’s journey toward zero harm: reducing postoperative respiratory failure. Physician Leadership Journal. 2023;10(3): 24–29.

[37] Escobar GJ, Liu VX, Schuler A, Lawson B, Greene JD, Kipnis P. Automated identification of adults at risk for in-hospital clinical deterioration. N Engl J Med. 2020;383(20):1951–1960. 33176085 10.1056/NEJMsa2001090 PMC7787261

[38] Mathis MR, Engoren MC, Williams AM, Prediction of postoperative deterioration in cardiac surgery patients using electronic health record and physiologic waveform data. Anesthesiology. 2022;137(5):586–601. 35950802 10.1097/ALN.0000000000004345 PMC10227693

