# Supplementary material for: A Least Absolute Shrinkage and Selection Operator-Derived Predictive Model for Postoperative Respiratory Failure in a Heterogeneous Adult Elective Surgery Patient Population
Source: CHEST Crit Care. Author manuscript; Available in PMC 2024 Mar 1. (PMC10907009; doi:10.1016/j.chstcc.2023.100025)
Supplement: 1 [file NIHMS1969099-supplement-1.docx]

**Supplementary Appendix for “A LASSO-derived predictive model for postoperative respiratory failure in a heterogeneous adult elective surgery patient population”**

**Table of Contents**

e-Table 1: Data Dictionary ………………………………………………………..……………………………………….………p2

e-Appendix 1: Structured Query Language Extract-Transform-Load Process and Sample Code ………………………….…….p10

e-Appendix 2: Secondary Analyses of Variable Selection and Model Performance…………………………….………….…….p20

e-Table 2: Coefficients of each Variable Fitted by LASSO Logistic Model for the Primary Model, Approach 1,

and Approach 2 ………………….…………………………………………………………………………………………….…..p23

e-Table 3: Predictive Model Performance of LASSO in the Primary Model using original data, with and without the optimism corrected procedure, Approach 1, and Approach 2.. ………..………………………………………..……….…………………..p28

e-Appendix 3: Comorbidity Sensitivity Analysis (with and without Elixhauser Comorbidity Variables)……….……………….p29

e-Table 4: Variables retained by the LASSO procedures in the logistic regression for predicting occurrence of postoperative respiratory failure……..…………………………………………………………………………………………….……………..p31

e-Table 5: Observed and optimism-corrected performance metrics for the LASSO logistic regression predicting post-operative respiratory failure with Elixhauser comorbidity variables…………………………………………………………….………..…p33

e-Figure 1: Receiver operating characteristic curve for fitted LASSO logistic regression predicting post-operative respiratory failure………………………………………………………………………………………………………………….…….…….p34

e-Figure 2: Precision-recall curve for fitted LASSO logistic regression predicting post-operative respiratory failure….……….p35

References ………………………………………………………………………………………………………………………...p36

**e-Table 1: Data Dictionary**

| **Variable Name** | **Variable Description** |
| --- | --- |
| BLINDED_IDN | Blinded ID # created for data analysis (to allow for removal of all PHI) |
| DERIVED_SURG_START | The derived surgery start time. Select anesthesia start time if it exists, otherwise select OR wheels in time as the surgery start time. |
| DERIVED_SURG_END | The derived surgery end time. Select anesthesia end time if it exists, otherwise select OR wheels out time as the surgery end time. |
| CASE_CONTROL | Flag to indicate whether the surgery is a postoperative respiratory failure patient or a non-PRF patient. If the surgical type is not elective , or the anesthesia type is not general, or patient wheels into the OR after 24 hours of admission, or patient was intubated at the time of wheels in, patient will be excluded from the cohort completely. Value is 1 if patient's total vent duration post operation (after OR wheels out time) is 48 hours or greater (PRF); otherwise, value is 0 if patient's is not intubated post operation or total vent duration post operation is less than 48 hours (non-PRF). |
| POD_DX | Post operative day number that patient has total post operative vent time of 48 hours or greater with surgery date = day 0. |
| PROCEDURE_NAME | The billing procedure name of the surgery. If ICD 10 billing procedure name is not null, then ICD 10 billing procedure name is selected. If ICD 10 billing procedure name is missing, then select ICD 9 billing procedure name. If both ICD 10 and ICD 9 billing procedure names are null, then select the CPT procedure name. |
| ADMISSION_DATE | The admission date/time of hospital admission of the surgery. |
| AGE | Patient's age at the time of admission. |
| SEX | Patient's sex at birth (sex as a biologic variable). Male; Female |
| RACE_BIN | Patient's mapped CDC race value. White; Black (African American or Black); Asian (Asian; Native Hawaiian or other Pacific Islander; American Indian or Alaska Native); Other (Other; Multiracial); Unknown (Unable to respond; Unavailable or unknown; Decline to State; Blank). |
| ETHNICITY_BIN | Patient's mapped CDC ethnicity value. Non-Hispanic; Hispanic; Unknown (Declined to state; Unavailable or Unknown; Blank) |
| PRIMARY_PAYOR_BIN | Medicare; Medicaid; Private/Commercial Insurance; Military/VA/Other; Unavailable/Unknown |
| ADMIT_DX_ICD_CODE | The billing admission ICD diagnosis code of the hospital admission. |
| ADMIT_DX_NAME | The diagnosis name of the billing admission diagnosis of the hospital admission. |
| SURG_SPECIALTY_NEWBIN | The surgical service that performs the surgery.  General Surgery; Cardiovascular Surgery; Neurosurgery, Spine; Oncology; Orthopedic; Urology, Gynecology; Head, Eyes, Ears, Nose, Throat (HEENT); Other  Based on Site Service Lines:  Cardiovascular: ACS, CVT, Cardiac Surgery, Cardiology, Peds CVT, Vascular  Ortho (non-spine): Ortho Foot & Ankle, Ortho Hand, Ortho Joint, Ortho Sports, Ortho Trauma, Orthopedics, Peds Orthopedics Neurosurgery, Spine: Neuro Spine, Neurosurgery, Ortho spine, Peds Neurosurgery  Oncology: Gynecologic Oncology, Ortho Oncology, Peds Hem Onc, Surgical Oncology HEENT: Dental, ENT, Ophthalmology Other: Pain management  General Surgery: GEN SURG, Colo-Rectal, FEMS (Bari/FG), Peds GI, Peds Surgery, Thoracic Surgery, Transplant, Plastics, Burn Urology and gynecology: Urogynecology, Urology, Obstetrics/GYN, Family Planning |
| DISCHARGE_DATE | The discharge date/time of the hospital admission. |
| DISCHARGE_DISP_GROUPER_NEWBIN | Home: Home Died: Death, Death - Hospice Readmit, Hospice, Hospice Readmit Other Facility: Acute Transfer, Long Term Acute Care, Psych, Rehab, SNF Other: AMA, Law Enforcement; Other |
| LOS_OBS | Hospital length of stay in days. |
| ICU_DAYS_OBS_ICUFILE | ICU length of stay in days. |
| ICD9_CODE | The primary billing ICD 9 procedure code. This is only available when Epic uses ICD 9 codes for billing. |
| ICD9_PROCEDURE_NAME | The primary billing ICD 9 procedure name. This is only available when Epic uses ICD 9 codes for billing. |
| ICD10_CODE | The primary billing ICD 10 procedure code. This is only available when Epic uses ICD 10 codes for billing. |
| ICD10_PROCEDURE_NAME | The primary billing ICD 10 procedure name. This is only available when Epic uses ICD 10 codes for billing. |
| CPT_CODE | The primary CPT code of the procedure. This is only available when surgery is billed using CPT code. |
| CPT_PROCEDURE_NAME | The primary CPT procedure name. This is only available when surgery is billed using CPT code. |
| HEIGHT_CM | Patient's most recent recorded height flowsheet value in centimeter prior to surgery. If there is no height recorded prior to surgery in the same admission, the height recorded closest to surgery post-surgery will be selected. |
| WEIGHT_KG | Patient's most recent recorded weight flowsheet value in kilogram prior to surgery. If there is no weight recorded prior to surgery in the same admission, the weight recorded closest to surgery post-surgery will be selected. |
| WEIGHT_IDEAL | The calculated ideal body weight in kilogram using the following formula by Dr. Devine: Ideal Body Weight (men) = 50kg + 2.3kg * (height_cm /2.54 - 60 ) Ideal Body Weight (women) = 45.5kg + 2.3kg *(height_cm/2.54 - 60 ). The original formula has height in inches. The above formula converts the height in centimeters to inches using height_in = height_cm/2.54. |
| BMI_CAT | The calculated numeric BMI category using the following formula: 0 = Underweight: < 18.5; 1 = Normal weight: 18.5 - 24.9; 2 = Overweight: 25 - 29.9; 3 = Obese1: 30 - 34.9; 4 = Obese2: 35 - 39.9; 5 = Morbidly obese: >40 |
| ASA_CLASS_BIN | The American Society of Anesthesiologists (ASA) rating category assigned by the anesthesiologist for the procedure. ASA class I and II; ASA class III, IV, V |
| VASO_YN | Flag to indicate whether any vasopressors are administered during the surgery (includes push dose and continuous infusion) |
| VASO_CONT_INF_YN | Flag to indicate whether any vasopressors are administered via continuous infusion during the surgery: 1=Yes; 0=No. If the administration route is continuous infusion or if the infusion rate is recorded, then the medication is considered as given continuous infusion. |
| VASO_CONT_INF_COUNT | The number of distinct medications (i.e., Phenylephrine, Epinephrine, Norepinephrine, etc.) that were given during the surgery via continuous infusion. |
| PHENYLEPHRINE_CONT_INF_YN | Flag to indicate whether Phenylephrine was given during the surgery via continuous infusion: 1=Yes; 0=No. |
| EPINEPHRINE_CONT_INF_YN | Flag to indicate whether Epinephrine was given during the surgery via continuous infusion: 1=Yes; 0=No. |
| NOREPINEPHRINE_CONT_INF_YN | Flag to indicate whether Norepinephrine was given during the surgery via continuous infusion: 1=Yes; 0=No. |
| VASOPRESSIN_CONT_INF_YN | Flag to indicate whether Vasopressin was given during the surgery via continuous infusion: 1=Yes; 0=No. |
| DOPAMINE_CONT_INF_YN | Flag to indicate whether Dopamine was given during the surgery via continuous infusion: 1=Yes; 0=No. |
| DOBUTAMINE_CONT_INF_YN | Flag to indicate whether Dobutamine was given during the surgery via continuous infusion: 1=Yes; 0=No. |
| MILRINONE_CONT_INF_YN | Flag to indicate whether Milrinone was given during the surgery via continuous infusion: 1=Yes; 0=No. |
| SURG_DATE | Date of the surgery. |
| ANES_START | Anesthesia start date/time. |
| ANES_END | Anesthesia end date/time. |
| ANES_DUR_60 | Duration of time from anesthesia start date/time to anesthesia end date/time in hours. |
| AIRWAY_START | Airway placement time. |
| AIRWAY_END | Airway removal time. |
| AIRWAY_PLACE | Categorical value for location of where the airway was placed: 1=Operating room; 2=Pre-Op or PACU (e.g., during perioperative time, but not in OR); 3=Preoperatively (e.g., ICU, Ward). |
| AIRWAY_PLACED_DEPARTMENT | The department unit type of where the airway was placed. |
| SURG_START | Surgery start time (i.e., incision start time). |
| SURG_END | Surgery end time (i.e., incision closed time). |
| SURG_DUR_60 | Duration of time from incision start time to incision closed time in hours. |
| MAP_MEDIAN | The median MAP value recorded during the surgery (from Derived Surgery Start to Derived Surgery End). |
| HEART_RATE_MEDIAN | The median heart rate recorded during the surgery (from Derived Surgery Start to Derived Surgery End). |
| SPO2_MEDIAN | The median SPO2 recorded during the surgery (from Derived Surgery Start to Derived Surgery End). |
| ETCO2_MEDIAN | The median ETCO2 recorded during the surgery (from Derived Surgery Start to Derived Surgery End). |
| VTCC_MEDIAN | The median VTCC recorded during the surgery (from Derived Surgery Start to Derived Surgery End). |
| RR_MEDIAN | The median RR recorded during the surgery (from Derived Surgery Start to Derived Surgery End). |
| FIO2_MEDIAN | The median FIO2 recorded during the surgery (from Derived Surgery Start to Derived Surgery End). |
| PEEP_MEDIAN | The median PEEP recorded during the surgery (from Derived Surgery Start to Derived Surgery End). |
| PIP_MEDIAN | The median PIP recorded during the surgery (from Derived Surgery Start to Derived Surgery End). |
| PLATEAU_PRESSURE_MEDIAN | The median Plateau Pressure recorded during the surgery (from Derived Surgery Start to Derived Surgery End). |
| OR_NET_FLUID_ML | Net fluid balance total, total fluid intake - total fluid output, during the surgery (from Derived Surgery Start to Derived Surgery End). |
| POST24_NET_FLUID_ML | Net fluid balance total, total fluid intake - total fluid output, in 24 hours post OR (from Derived Surgery End to 24 hours after Derived Surgery End). |
| TOTAL_MORPHINE_MG | Total oral morphine equivalent dose in milligram based on calculation in Epic. |
| COMORBIDITY_SCORE | Total Elixhauser comorbidity score. The total score is calculated from the 31 comorbidity categories. Score for each of the comorbidities can be blank, a positive integer or a negative integer. |
| COMORBIDITY_COUNT | Number of Elixhauser comorbidity categories. |
| CM_01_AIDS_SCORE | Elixhauser comorbidity subscore: AIDS HIV. The weighted subscore for this category is 0. |
| CM_02_ALCOHOL_SCORE | Elixhauser comorbidity subscore: ALCOHOL ABUSE. The weighted subscore for this category is 0. |
| CM_03_BLANE_SCORE | Elixhauser comorbidity subscore: BLOOD LOSS ANEMIA. The weighted subscore for this category is -2. |
| CM_04_CARIT_SCORE | Elixhauser comorbidity subscore: CARDIAC ARRYTHMIAS. The weighted subscore for this category is 5. |
| CM_05_CHF_SCORE | Elixhauser comorbidity subscore: CONGESTIVE HEART FAILURE. The weighted subscore for this category is 7. |
| CM_06_COAG_SCORE | Elixhauser comorbidity subscore: COAGULOPATHY. The weighted subscore for this category is 3. |
| CM_07_CPD_SCORE | Elixhauser comorbidity subscore: CHRONIC PULMONARY DISEASE. The weighted subscore for this category is 3. |
| CM_08_DANE_SCORE | Elixhauser comorbidity subscore: DEFICIENCY ANEMIA. The weighted subscore for this category is -2. |
| CM_09_DEPRE_SCORE | Elixhauser comorbidity subscore: DEPRESSION. The weighted subscore for this category is -3. |
| CM_10_DIABC_SCORE | Elixhauser comorbidity subscore: DIABETES COMPLICATED. The weighted subscore for this category is 0. |
| CM_11_DIABUNC_SCORE | Elixhauser comorbidity subscore: DIABETES UNCOMPLICATED. The weighted subscore for this category is 0. |
| CM_12_DRUG_SCORE | Elixhauser comorbidity subscore: DRUG ABUSE. The weighted subscore for this category is -7. |
| CM_13_FED_SCORE | Elixhauser comorbidity subscore: FLUID ELECTROLYTE DISORDERS. The weighted subscore for this category is 5. |
| CM_14_HYPC_SCORE | Elixhauser comorbidity subscore: HYPERTENSION COMPLICATED. The weighted subscore for this category is 0. |
| CM_15_HYPOTHY_SCORE | Elixhauser comorbidity subscore: HYPOTHYROIDISM. The weighted subscore for this category is 0. |
| CM_16_HYPUNC_SCORE | Elixhauser comorbidity subscore: HYPERTENSION UNCOMPLICATED. The weighted subscore for this category is 0. |
| CM_17_LD_SCORE | Elixhauser comorbidity subscore: LIVER DISEASE. The weighted subscore for this category is 11. |
| CM_18_LYMPH_SCORE | Elixhauser comorbidity subscore: LYMPHOMA. The weighted subscore for this category is 9. |
| CM_19_METACANC_SCORE | Elixhauser comorbidity subscore: METASTATIC CANCER. The weighted subscore for this category is 12. |
| CM_20_OBES_SCORE | Elixhauser comorbidity subscore: OBESITY. The weighted subscore for this category is -4. |
| CM_21_OND_SCORE | Elixhauser comorbidity subscore: OTHER NEUROLOGICAL DISORDERS. The weighted subscore for this category is 6. |
| CM_22_PARA_SCORE | Elixhauser comorbidity subscore: PARALYSIS. The weighted subscore for this category is 7. |
| CM_23_PCD_SCORE | Elixhauser comorbidity subscore: PULMONARY CIRCULATION DISORDERS. The weighted subscore for this category is 4. |
| CM_24_PSYCHO_SCORE | Elixhauser comorbidity subscore: PSYCHOSES. The weighted subscore for this category is 0. |
| CM_25_PUD_SCORE | Elixhauser comorbidity subscore: PEPTIC ULCER DISEASE. The weighted subscore for this category is 0. |
| CM_26_PVD_SCORE | Elixhauser comorbidity subscore: PERIPHERAL VASCULAR DISORDERS. The weighted subscore for this category is 2. |
| CM_27_RF_SCORE | Elixhauser comorbidity subscore: RENAL FAILURE. The weighted subscore for this category is 5. |
| CM_28_RHEUMD_SCORE | Elixhauser comorbidity subscore: RHEUMATOID ARTHRITIS/COLLAGEN VASCULAR DISEASES. The weighted subscore for this category is 0. |
| CM_29_SOLIDTUM_SCORE | Elixhauser comorbidity subscore: SOLID TUMOR WITHOUT METASTASIS. The weighted subscore for this category is 4. |
| CM_30_VALV_SCORE | Elixhauser comorbidity subscore: VALVULAR DISEASE. The weighted subscore for this category is -1. |
| CM_31_WLOSS_SCORE | Elixhauser comorbidity subscore: WEIGHT LOSS. The weighted subscore for this category is 6. |
| DEATH_DATE | The date that patient died. |
| ANESTHESIA_TYPE | The primary anesthesia type used for this surgical case. |
| OR_PHASE_COUNT | The number of OR ADT metaphase(s) of this admission (i.e., the number of times patient went to the OR). |
| ADM_TO_OR_START_HRS_GROUP | The category group for the number of hours from admission to OR wheels in time (i.e. ≤24). |
| ADT_NON_PACU_DEPT_POST | The department unit name of where patient is located immediately post OR (excluding PACU). |
| ADT_NON_PACU_DEPT_TYPE | The department unit type of where patient is located immediately post OR (excluding PACU). |
| ANESTHESIA_AIRWAY_TYPE | Airway type documented in the Anesthesia record. |
| FIRST_O2_DEVICE_POST_OR | First oxygen device type recorded immediately after OR wheels out time. |
| MV_DURATION_IN_OR_HRS | The number of hours that patient was intubated while in the OR between derived surgery start time to derived surgery end time. |
| REINTUBATED_POST_OR | Flag to indicate whether patient was reintubated post OR: 1=Yes; 0 or blank=No. Reintubation flag if there is a mechanical ventilator metaphase after airway removal time. |
| MV_POST_OR_MP_DURATION_HRS | The duration in hours of the first continuous mechanical ventilator metaphase post OR. |
| MV_POST_MAX_MP_DURATION_HRS | The longest duration in hours of the continuous mechanical ventilator metaphase post OR. |
| MV_POST_OR_DURATION_HRS | The total duration in hours of all continuous mechanical ventilator metaphase(s) post OR. |
| TIME_TO_REINTUBATION_HRS | Time in hours from airway removal time to start time of first mechanical ventilator metaphase post OR. |
| HAS_TRACHEOSTOMY_AT_ADMSN | Flag to indicate whether patient has a Tracheostomy airway at the time of hospital admission. |
| ICD9_CCS_GroupName | ICD9 Procedure code rolled up into bins by HCUP CCS (<https://hcup-us.ahrq.gov/toolssoftware/ccs/ccs.jsp>) |
| ICD10_CCS_LevelOne | ICD10 Procedure code rolled up into bins by HCUP CCS (<https://hcup-us.ahrq.gov/toolssoftware/ccsr/ccs_refined.jsp>) |
| ICD_NEW_BIN | NOTE: CREATED ONE ICD NEW BIN that combines the ICD9 and ICD10 data into like bins |
| CPT_CCS_GroupName | CPT code rolled up into bins by HCUP CCS (<https://hcup-us.ahrq.gov/toolssoftware/ccs_svcsproc/ccssvcproc.jsp>) |

**e-Appendix 1:** **Structured Query Language Extract-Transform-Load Process and Sample Code for Mechanical Ventilation Metaphase >48 hours from Operating Room Departure through Hospital Discharge**

**Introduction:**

Here we describe a single site proof-of-concept study using an automated structured query language (SQL)-based extract, transform, and load (ETL) procedure that enables rapid acquisition of data exclusively from our electronic health record (EHR), which uses the Epic platform (https://www.epic.com). We used the curated and validated data to develop a predictive model^1^ for PRF following elective surgery in adults. We report our findings following STROBE and TRIPOD guidelines.^2,3^ We hypothesized that our predictive model would have at least good discrimination and would be well-calibrated across its range of predicted probabilities. We anticipate that our standardized, automated data acquisition and model development methods will allow us to expand our SQL ETL process across the five centers of our University of California Critical Care Research Collaborative (UC^3^RC) for further model development and validation. Generating and critically evaluating standardized, automated approaches to large-scale multicenter research using real-world data is crucial in predictive modeling of rare adverse events, such as PRF.

**Methods:**

Data Extract, Transform, and Load (ETL) Procedure

Our team developed logic to enable all variables of interest to be curated from discrete data fields. We excluded emergency surgery in our model, although we are aware many predictive models include emergency surgery. Inclusion and exclusion criteria are listed in our main paper. We defined postoperative respiratory failure (PRF) as >48 hours of cumulative mechanical ventilation following the index elective surgical procedure. This derived timeframe started from the documented anesthesia end time (or the documented time of wheels out of the operating room [OR] for the 44 surgical encounters for which there was an anesthesia start time, but the anesthesia end time was missing) through the documented hospital discharge time. As our goal was to develop a predictive model that could be used to identify patients at high risk for PRF and make postoperative level of care and intervention decisions, predictors of interest spanned the pre- and intra-operative continuum. These included demographics (age, sex, race, ethnicity, primary payer), preexisting comorbidities (American Society of Anesthesiologist (ASA) Classification System, Elixhauser Comorbidity count and score^4,5^), and perioperative factors (anesthesia duration, surgical procedure and specialty, vital signs, intraoperative ventilator and medication management, and OR fluid balance) (complete data dictionary provided in e-Table 1). We utilized SQL coding (sample below) to perform the data ETL procedure from our Epic EHR. Through an iterative process, two clinicians validated every aspect of data curation by comparing the SQL ETL output for every patient with PRF and a random 10% of patients without PRF to the criterion standard of manual chart review. Interrater reliability was not empirically measured, but we continued this process until there was 100% agreement between the two clinician reviewers. While this process was time-intensive, our team felt the scalability of automated data curation was a substantial improvement over our prior work with manual data curation, and it should enable extension to other health systems that use the Epic EHR with minimal adaptation.

Missing Data:

Only variables with <2.5% missing data were considered for model development. Missing data were imputed to the mode (categorical variables) or median (continuous variables) for the cohort. Using this approach, the only variable of interest we were unable to include in the least absolute shrinkage and selection operator (LASSO) variable selection was ventilator plateau pressure, which was only documented for six patients with PRF and 26 patients without PRF. While others have included preoperative lab values in their models, despite also having over 50% missing data,^6^ we opted not to consider preoperative labs values in our elective surgery population due to the high percentage of missing data. In our sample the percentage of missing lab data was as follows: (1) albumin 71.1% missing in patients with PRF, 72.5% missing in patients without PRF; (2) creatinine 60.0% missing in patients with PRF, 59.9% missing in patients without PRF; (3) hemoglobin 60.0% missing in patients with PRF, 58.6% missing in patients without PRF. Our SQL logic included lab values reported within four months prior to surgery.

**Sample Code:**

**oxygen_device_metaphase_from_clarity.sql**

/*

Purpose: This script pulls oxygen device metaphase data from Clarity.

NOTE: Derived values for oxygen devices must set up in a custom table GEN_FLOWSHEET_ATTR

*/

-- replace CSN ID

define pat_enc_csn_id = 200000802942

;

with o2_device as (

-- flowsheet mapping values and priority

-- this is site specific and need to be set up based on values for oxygen device, mode, invasive mode, start vent and stop vent flowsheets

select

vent.attribute_value oxygen_device_meas,

gen_flowsheet_attr.attribute_value_2 oxygen_device_phase,

gen_flowsheet_attr.flo_meas_id,

gen_flowsheet_attr.meas_value,

gen_flowsheet_attr.disp_name,

gen_flowsheet_attr.attribute_order process_priority

from

sa_covid.gen_flowsheet_attr vent

inner join sa_covid.gen_flowsheet_attr on vent.registry_name = gen_flowsheet_attr.registry_name

and vent.flo_meas_id = gen_flowsheet_attr.flo_meas_id

where

vent.registry_name = 'ADULT REGISTRY'

and vent.attribute_code = 'VENTILATION FLOWSHEET'

and gen_flowsheet_attr.attribute_code = 'OXYGEN DEVICE'

and gen_flowsheet_attr.attribute_value is not null

)

,

-- pull flowsheet values and order the priority

flwsht as (

select

pat_enc_csn_id,

hosp_admsn_time,

hosp_disch_time,

flo_meas_id,

disp_name,

oxygen_device_phase,

recorded_time,

lag(oxygen_device_phase) over (partition by pat_enc_csn_id order by recorded_time) previous_phase,

lead(oxygen_device_phase) over (partition by pat_enc_csn_id order by recorded_time) next_phase

from (

select /*+ DRIVING_SITE(ip_flwsht_rec) */

pat_enc_hsp.pat_enc_csn_id,

pat_enc_hsp.hosp_admsn_time,

pat_enc_hsp.hosp_disch_time,

ip_flwsht_meas.flo_meas_id,

ip_flo_gp_data.disp_name,

ip_flwsht_meas.recorded_time,

ip_flwsht_meas.meas_value,

o2_device.oxygen_device_phase,

row_number() over (partition by pat_enc_hsp.pat_enc_csn_id, ip_flwsht_meas.recorded_time order by o2_device.process_priority) rec_no

from

pat_enc_hsp

inner join ip_flwsht_rec on pat_enc_hsp.inpatient_data_id = ip_flwsht_rec.inpatient_data_id

inner join ip_flwsht_meas on ip_flwsht_rec.fsd_id = ip_flwsht_meas.fsd_id

inner join ip_flo_gp_data on ip_flwsht_meas.flo_meas_id = ip_flo_gp_data.flo_meas_id

inner join o2_device on ip_flwsht_meas.flo_meas_id = o2_device.flo_meas_id

and ';' || ip_flwsht_meas.meas_value || ';' like o2_device.meas_value

where

1=1

and pat_enc_hsp.pat_enc_csn_id = &pat_enc_csn_id

and pat_enc_hsp.hosp_admsn_time <= ip_flwsht_meas.recorded_time

and pat_enc_hsp.hosp_disch_time >= ip_flwsht_meas.recorded_time

-- Site specific flow measure ID

and ip_flwsht_meas.flo_meas_id in (

'301528' -- Mode

, '805370' -- Device (Oxygen Therapy)

, '303805' -- Non-invasive

, '303675' -- Invasive Ventilation Started

, '303686' -- Invasive Ventilation Stopped

)

and ip_flwsht_meas.isaccepted_yn = 'Y'

) where rec_no = 1

)

--select * from flwsht;

,

-- set the phases of each derived oxygen device phase

phase as (

select

pat_enc_csn_id,

hosp_admsn_time,

hosp_disch_time,

oxygen_device_phase,

recorded_time,

time_flag,

-- lag(recorded_time) over (partition by pat_enc_csn_id order by recorded_time) previous_phase_time,

lead(recorded_time) over (partition by pat_enc_csn_id order by recorded_time) next_phase_time

from (

select

pat_enc_csn_id,

hosp_admsn_time,

hosp_disch_time,

oxygen_device_phase,

recorded_time,

case

when (previous_phase is null

or previous_phase <> oxygen_device_phase)

and (next_phase is null

or next_phase <> oxygen_device_phase)

then 'START/END'

when previous_phase is null

or previous_phase <> oxygen_device_phase

then 'START'

when next_phase is null

or next_phase <> oxygen_device_phase

then 'END'

else

null

end time_flag

from

flwsht

)

where time_flag in ('START', 'START/END')

)

-- create the metaphase with oxygen delivery device, start and end time

select

pat_enc_csn_id,

hosp_admsn_time,

hosp_disch_time,

oxygen_device_phase,

recorded_time start_time,

coalesce(next_phase_time - 1/1440, hosp_disch_time) end_time,

round((coalesce(next_phase_time - 1/1440, hosp_disch_time) - recorded_time) * 1440,0) + 1 duration_minutes

from phase

order by start_time

;

**Discussion:**

In contrast to our previous PRF research that used manual chart abstraction,^7-9^ our current study developed and validated an automated ETL process to enable efficient, standardized acquisition of real-world data from our EHR. We focused on discrete data fields within our EHR and chose not to utilize natural language processing to query data from narrative notes. We also developed logic to support derived clinical concepts that we hypothesized would be important predictors of PRF, such as OR fluid balance and the Elixhauser comorbidity count and score. The potentially extensible nature of SQL-based ETL processes should allow adaptation of our methods to the EHRs of other research sites, thereby enabling rapid data acquisition and large-scale research into rare events like PRF that would not be feasible if data collection were restricted to manual chart review.

While our health system, like others, has used SQL ETL processes for clinical data, this predictive model represents our first use of this method for perioperative flowsheet data from the Epic OpTime module. The SQL ETL process provides a foundational framework for scalability to the other four major academic medical centers that comprise our health system, all of which are on similar versions of the Epic EHR platform. At each center, data from the Epic Chronicles EHR, used to record and access clinical data in real-time, are stored in Epic Clarity, a highly granular, comprehensive relational database designed for clinical and operational reporting and data analysis. Whereas the Chronicles data model is optimized for efficient data retrieval from an individual patient’s record, Clarity is designed to efficiently retrieve data from large populations of patient records. The complexity of the Clarity data model, the granularity of the data within it, and the heterogeneity of clinical documentation frequently require the development of additional ETL procedures - like those developed in this study - to enable acquisition of data in the form of standardized, curated concepts. For example, while oxygen delivery device data are recorded minute-by-minute for every patient throughout the hospital encounter, mechanical ventilation start and stop times, and thus total duration, are not consistently recorded thus requiring additional ETL procedures to acquire accurate data from Epic Clarity, as others have done for the Cerner platform.^10-12^

**Diagram of Extract-Transform-Load Process:**


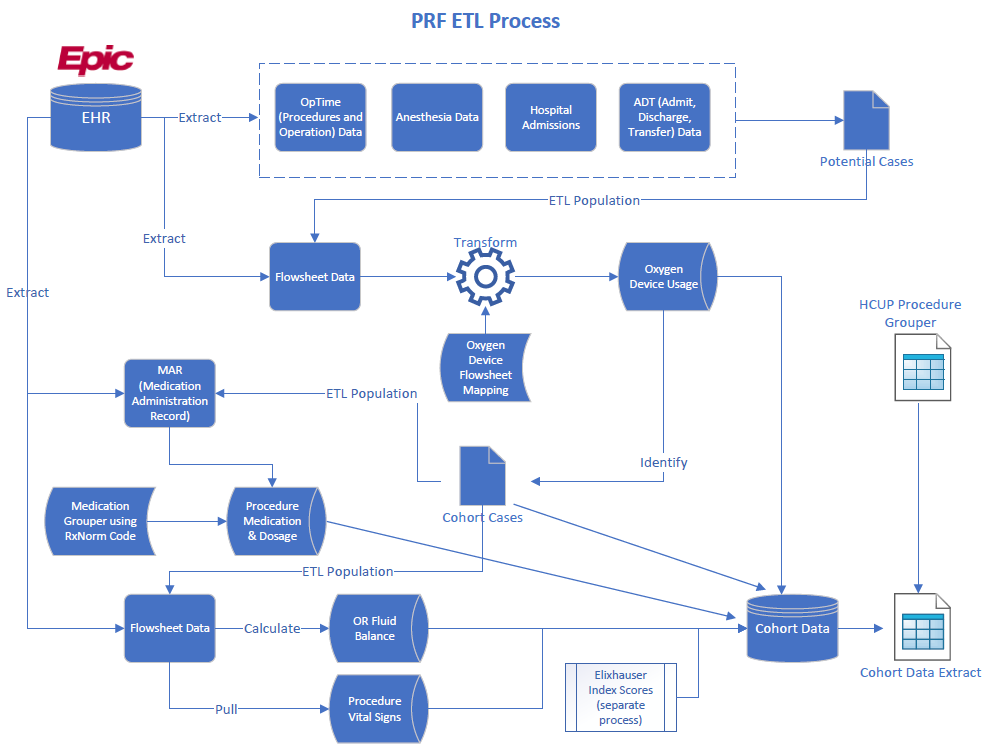


We have provided a sample of our SQL code to curate cumulative invasive mechanical ventilation (MV) from Clarity and derive our outcome variable of “MV >48 hours” (our full SQL code set is available upon request). Preliminary review of the EHRs from other centers in our health system indicates that documentation of MV occurs in a similar manner across centers, suggesting extensibility of our methods to health systems using comparable versions of Epic. The next phase of our work will be to apply our SQL code to other sites within our health system to curate a dataset and evaluate the external validity of our predictive model. While clinical documentation is known to vary between health systems, other efforts both within^13^ and outside^14^ our health system suggest that our approach will generalize with some degree of site-specific adaptation. Future research will need to identify generalizable principles to guide site-specific ETL adaptation between Epic sites, across EHR vendor-specific data models, and compared to use of less comprehensive albeit more standardized vendor-agnostic data models.^15^ If successfully extended to these other centers, our approach will permit external validation of our PRF predictive model and will further support the use of extensible ETL procedures as a research strategy to enable large-scale studies of rare events using real-world data that would otherwise be cost-prohibitive through either traditional manual chart review or site-specific, information technology-dependent data curation and provisioning services.

**e-Appendix 2: Secondary Analyses of Variable Selection and Model Performance**

Assessment of Primary Model

The available data set was strongly imbalanced with 99.1% of the patients not experiencing PRF. Due to the small number of patients with PRF, we used the entire data set to construct a predictive model to maximize the available number of patients with PRF and to construct a model appropriate for the prevalence of the outcome in clinical settings. Because performance metrics will be optimistic (e.g., high) when evaluated for the data on which a model is trained, we employed a bootstrap procedure to correct for this optimism in the performance metrics estimated on the data set used to derive the model. Further, variable selection in model building can be unstable, changing with the sample used to construct the model. As a secondary analysis to evaluate the robustness of the performance estimates and variable selection found in the primary analysis, we conducted additional model building and assessment.

**Methods**

For this secondary analysis, we temporally split the data into a training set (2014-2018) and a test set (2019-2021) yielding a training set of 15,824 (PRF = 135, non-PRF = 15,689) patients and a test set of 8,175 patients (PRF = 90, non-PRF = 8,085). We then used two approaches to construct models with the training set and estimated performance in the hold-out test set.

In Approach 1, we fit a LASSO logistic regression to the training set following the same procedure we used in the Primary Analysis (described in the main paper). Variables with non-zero coefficients from the LASSO procedure were used to construct a logistic regression model and performance of the model was evaluated on the test set.

In Approach 2, using just the training set, we balanced patients with PRF (n = 135) and patients without PRF by randomly selecting an equal number of patients without PRF and fitting the LASSO logistic regression to the balanced data set. This procedure was repeated 1,000 times with random selections of 135 patients without PRF, but with retention of all patients with PRF. LASSO logistic regressions were applied to each of the 1,000 balanced data sets yielding 1,000 models. Because the variables retained in the logistic regression by the LASSO procedure could vary among the 1,000, this procedure provided an assessment of the stability of the variable selection. A final model was constructed based on the Approach 2 results using predictors retained in at least 50% of the 1,000 models and evaluated on the test set.

**Results**

The predictor variables retained in models fitted under Approach 1 were similar to those retained in the Primary Model with Approach 1 retaining 15 of the 18 predictors in the Primary Model. Anesthesia duration (hours), ASA ≥III, and vasopressors were the only variables retained in at least 50% of the models under Approach 2. These 3 variables were also retained in Approach 1 and were also selected in >95% of the bootstrap models in the Primary Analysis (e-Table 2). These results suggest that these three variables are robust predictors of PRF.

Estimates of performance metrics for the Primary Model, with and without optimism correction, and for models developed through Approaches 1 and 2 applied to the hold-out test set are shown in e-Table 3. The performance metrics for Approaches 1 and 2, when applied to the hold-out test sets, were slightly worse than those for the Primary Model evaluated on the full data set. Specifically, the estimated area under the receiver operating characteristic curve (AUC) decreased from 0.835 in the Primary Model with optimism correction to 0.786 and 0.763 for Approach 1 and 2, respectively in the supplementary analyses but the 95% confidence intervals for these estimates overlapped. On the other hand, the area under the precision-recall curve (AUPRC) values increased from 0.156 to 0.172 for Approach 1 but decreased to 0.059 for Approach 2 with overlapping 95% confidence intervals between Approach 1 and the Primary Model but not for Approach 2. These results generally confirm the robustness of optimism-corrected performance findings for the Primary Model.

**e-Table 2: Coefficients of each Variable Fitted by LASSO Logistic Model for the Primary Model, Approach 1, and Approach 2**

|  | **Predictor Variable** | **Coefficient^a^** | **Probability of Selection^b^**  **(%)** | **Coefficient Approach 1^c^** | **Coefficient Approach 2^c^** | **Variable Variability**  **(%)^d^** |
| --- | --- | --- | --- | --- | --- | --- |
| 1 | intercept | -7.44 | 100 | -7.46 | -6.48 | 100 |
| 2 | ASA Class III or greater^e^ | 0.57 | 100 | 0.45 | 0.39 | 78.8 |
| 3 | Anesthesia duration (per hour) | 0.19 | 100 | 0.21 | 0.25 | 100 |
| 4 | Vasoactive medication in the operating room | 0.29 | 95.6 | 0.33 | 0.19 | 50.3 |
| 5 | Operations on the digestive system | 0.11 | 88.8 | 0.02 | NA ^f^ | 1.7 |
| 6 | Operations by an orthopedic surgeon (non-spine) | -0.22 | 82.8 | -0.03 | NA | 30 |
| 7 | Medicare (as the primary payer) | 0.47 | 100 | 0.16 | NA | 4.1 |
| 8 | Operations on the urinary system | 0.17 | 81.2 | - ^g^ | NA | 0.2 |
| 9 | Body Mass Index category 3 (Obese1: 30 – 34.9) | -0.07 | 80.4 | -0.10 | NA | 4.8 |
| 10 | Net Fluid at end of operating room procedure (per liter) | 0.17 | 100 | 0.21 | NA | 7.3 |
| 11 | Operations on the cardiovascular system | 0.92 | 100 | 0.67 | NA | 22.9 |
| 12 | Fraction of inspired oxygen (FiO2) (median) | 0.01 | 98 | 0.01 | NA | 14.2 |
| 13 | Operations on the neurologic system | 0.55 | 96.4 | 0.07 | NA | 0.4 |
| 14 | Age (years, per year) | 0 | 59.6 | - | NA | 1.6 |
| 15 | Heart rate (median) | 0.009 | 94.8 | 0.003 | NA | 3.6 |
| 16 | Tidal volume set (median) | -0.001 | 92.4 | -0.001 | NA | 2.6 |
| 17 | Operations on the musculoskeletal system | -0.40 | 91.2 | -0.57 | NA | 46.4 |
| 18 | Body Mass Index category 4 (Obese2: 35 - 39.9) | 0 | 49.2 | - | NA | 0.3 |
| 19 | Positive end expiratory pressure (median) | 0 | 49.2 | 0.03 | NA | 1.2 |
| 20 | Primary payer Medi-Cal | 0.19 | 82.4 | 0.11 | NA | 3.7 |
| 21 | Mean arterial pressure (median) | 0 | 45.6 | - | NA | 0.1 |
| 22 | Ethnicity not Hispanic or Latino | 0 | 43.2 | - | NA | 0.4 |
| 23 | End tidal carbon dioxide (median) | -0.01 | 75.6 | - | NA | 0.7 |
| 24 | Operations by a general surgeon | 0 | 71.6 | - | NA | 0.6 |
| 25 | Race Unknown | 0.26 | 62.8 | - | NA | 0.3 |
| 26 | Peak inspiratory pressure (median) | 0 | 62.8 | - | NA | 0.4 |
| 27 | Respiratory rate (median) | 0 | 38.8 | - | NA | 0.3 |
| 28 | Morphine total dose in OR | 0 | 56.4 | 0.00003 | NA | 0.3 |
| 29 | Operations on the female genital organs | 0 | 54.8 | -0.09 | NA | 5.0 |
| 30 | Ethnicity unknown | 0 | 50.8 | 0.04 | NA | 1.8 |
| 31 | Operations on the respiratory system | 0 | 29.6 | - | NA | 0.9 |
| 32 | Race Black | 0 | 28.8 | - | NA | 0.3 |
| 33 | Sex (as a biologic variable) Male | 0 | 48 | - | NA | 0.1 |
| 34 | Operations by a neurosurgeon (inc. spine) | 0 | 28 | - | NA | 0.2 |
| 35 | Operations by a HEENT surgeon | 0 | 26.8 | - | NA | 0.3 |
| 36 | Operations on the ear | 0 | 42.8 | - | NA | 0.6 |
| 37 | Race White | 0 | 40.8 | - | NA | 0.5 |
| 38 | Operations on the nose, mouth, and pharynx | 0 | 40.4 | - | NA | 0.4 |
| 39 | Body Mass Index category 1 (Normal: 18.5 – 24.9) | 0 | 38.8 | - | NA | 0.5 |
| 40 | Operations on the male genital organs | 0 | 25.2 | - | NA | 0.8 |
| 41 | Operations by a urology and gynecology surgeon | 0 | 34 | - | NA | 0.1 |
| 42 | SpO2 (median) | 0 | 34 | - | NA | 0.2 |
| 43 | Body Mass Index category 2 (Overweight: 25 – 29.9) | 0 | 33.2 | - | NA | 0.2 |
| 44 | Operations on the hemic and lymphatic system | 0 | 20.8 | - | NA | 0.3 |
| 45 | Operation by a surgeon otherwise not listed | 0 | 0 | - | NA | 0.2 |
| 46 | Primary payer unknown | 0 | 28 | - | NA | 0 |
| 47 | Operations on the endocrine system | 0 | 25.6 | - | NA | 0.4 |
| 48 | Body Mass Index category 5 (Morbidly Obese: >40) | 0 | 25.2 | - | NA | 0.2 |
| 49 | Operation by an oncology surgeon | 0 | 26.4 | - | NA | 0.2 |
| 50 | Operations on the eye | 0 | 26 | - | NA | 0.1 |
| 51 | Operations on the integumentary system | 0 | 22.4 | - | NA | 0.3 |
| 52 | Race Other | 0 | 21.2 | - | NA | 0 |
| 53 | Operations on the obstetrical system | 0 | 23.2 | - | NA | 0 |

^a^ Coefficient is the predictor variable coefficient of LASSO for the Primary Model (that is described in the main paper).

^b^ Probability of Selection is the percentage of bootstrap samples in the Primary Analysis for which the variable was retained (i.e., had a non-zero coefficient)

^c^ Coefficient of Approach 1 and Approach 2 are variable coefficients of LASSO logistic regression as described in the supplemental text.

^d^ Variable variability is defined as the percentage of 1,000 resampled datasets under Approach 2 in which the variable was retained by the LASSO logistic regression procedure. Both Probability of Selection and Variable Variability measures provide an assessment of the stability of the variable selection.

^e^ American Society of Anesthesiologists (ASA) Classification System

^f^ For Approach 2, NA indicates that the variable was not included in the final models developed using variables retained in at least 50% of the 1,000 models.

^g^ Empty values (denoted as “-“) for coefficients indicate the variable was not selected.

**e-Table 3: Predictive Model Performance of LASSO in the Primary Model using original data, with and without the optimism corrected procedure, and Approach 1, and Approach 2**

|  | **Sensitivity**  **(95% CI)** | **Specificity**  **(95% CI)** | **PPV^a^**  **(95% CI)** | **NPV^b^**  **(95% CI)** | **AUC^c^**  **(95% CI)** | **AUPRC^d^**  **(95% CI)** |
| --- | --- | --- | --- | --- | --- | --- |
| Primary Model (without optimism correction) | 0.693 (0.638, 0.758) | 0.858 (0.851, 0.86) | 0.042 (0.037, 0.05) | 0.997 (0.996, 0.997) | 0.851 (0.824, 0.878) | 0.174 (0.123, 0.221) |
| Primary Model (with optimism correction) | 0.647 (0.593, 0.713) | 0.858  (0.851, 0.86) | 0.042  (0.035, 0.048) | 0.996  (0.995, 0.996) | 0.835  (0.808, 0.862) | 0.156  (0.105, 0.203) |
| Approach 1 | 0.678 (0.581, 0.774) | 0.766 (0.757, 0.775) | 0.031 (0.024, 0.039) | 0.995 (0.994, 0.997) | 0.786 (0.733, 0.838) | 0.172 (0.094, 0.249) |
| Approach 2 | 0.611 (0.51, 0.712) | 0.796 (0.787, 0.805) | 0.032 (0.024, 0.041) | 0.995 (0.993, 0.996) | 0.763 (0.711, 0.815) | 0.059 (0.011, 0.108) |

^a^ Positive predictive value

^b^ Negative predictive value

^c^ Area under the receiver operating characteristic curve

^d^ Area under the precision-recall curve

**e-Appendix 3: Comorbidity Sensitivity Analysis**

Comorbid conditions could influence a patient’s risk of PRF. However, accurate curation, from discrete data fields, of comorbidities that were present on admission often requires access to administrative billing coding information that is only available at discharge. While comorbidity information is often included in a preoperative history and physical, the documentation is located within the narrative note, not in discrete fields, and therefore not amenable to SQL ETL procedures. While comorbidity information does exist in discrete fields in the provider-generated “problem list” function in Epic, we have found the problem list is often not fully updated. We sought to determine the impact of Elixhauser comorbidity count and score on the model and its performance. We hypothesized that model performance with Elixhauser data would not be significantly improved when compared to model performance without Elixhauser data. Further, we hypothesized that a model inclusive of Elixhauser would not only be more complex to utilize, but it would also preclude use of the predictive model at the end of surgery to predict risk of PRF.

Methods

As a sensitivity analysis we repeated the primary model building with inclusion of the Elixhauser comorbidity score and a count of the total number of comorbidities.

Results

When compared to our primary model, the resultant 13-variable predictive model (e-Table 4) had a negligible increase in optimism corrected AUC from 0.835 to 0.84 and in AUPRC from 0.156 to 0.162 (e-Table 5, e-Figure 1, e-Figure 2) when compared to the primary model.

**e-Table 4:** Variables retained by the LASSO procedures in the logistic regression for predicting occurrence of postoperative respiratory failure.

| **Predictor Variable** | **Coefficient** | **Probability Selected (%)^a^** |
| --- | --- | --- |
| (intercept) | -7.4129 | 100 |
| Anesthesia duration (per hour) | 0.1863 | 100 |
| Net Fluid at end of the operation (per liter) | 0.1676 | 100 |
| Operations on the cardiovascular system | 0.8026 | 100 |
| Elixhauser Comorbidity Count^b^ (per comorbidity) | 0.0749 | 99.6 |
| Elixhauser Comorbidity Score^b^ (per point) | 0.0306 | 98.8 |
| Medicare (as the primary payer) | 0.1611 | 95.6 |
| Fraction of inspired oxygen (FiO2) (median) | 0.0068 | 94.8 |
| Operations on the nervous system | 0.3291 | 92 |
| Operations on the musculoskeletal system | -0.3480 | 91.6 |
| Vasoactive medication in the operating room | 0.1374 | 90.8 |
| ASA Class >III | 0.1200 | 88 |
| Tidal volume (median) | -0.0001 | 83.2 |
| Heart rate (median) | 0.0005 | 81.2 |

^a^ Probability selected is the percentage of bootstrap samples in which the variable was retained.

^b^ Comorbid conditions included in the Elixhauser: congestive heart failure, cardiac arrythmias, valvular disease, pulmonary circulatory disorders, peripheral vascular disorders, hypertension (uncomplicated), hypertension (complicated), paralysis, other neurological disorders, chronic pulmonary disease, diabetes (uncomplicated), diabetes (complicated), hypothyroidism, renal failure, liver disease, peptic ulcer disease excluding bleeding, AIDS/HIV, lymphoma, metastatic cancer, solid tumor without metastasis, rheumatoid arthritis/collagen vascular diseases, coagulopathy, obesity, weight loss, fluid and electrolyte disorders, blood loss anemia, deficiency anemia, alcohol abuse, drug abuse, psychoses, depression.^4^ Elixhauser comorbidity score is calculated by assigning weights to each comorbidity based on van Walraven et al.^5^

**e-Table 5:** Observed and optimism-corrected performance metrics for the LASSO logistic regression predicting post-operative respiratory failure with Elixhauser comorbidity variables

|  | Sensitivity  (95% CI) | Specificity  (95% CI) | PPV^a^  (95% CI) | NPV^b^  (95% CI) | AUC^c^  (95% CI) | AUPRC^d^  (95% CI) | Brier Score |
| --- | --- | --- | --- | --- | --- | --- | --- |
| Observed Performance (original data performance)^e,f^ | 0.702 (0.642, 0.762) | 0.861 (0.857, 0.866) | 0.046 (0.039, 0.053) | 0.997 (0.996, 0.998) | 0.854 (0.827, 0.881) | 0.179 (0.127, 0.227) | 0.008 |
| Optimism Corrected Performance | 0.66 (0.601, 0.72) | 0.861  (0.856, 0.865) | 0.043  (0.036, 0.05) | 0.996  (0.995, 0.997) | 0.84  (0.813, 0.868) | 0.162  (0.11, 0.209) | 0.009 |
| Estimated Optimism | 0.042 | 0 | 0.002 | 0 | 0.013 | 0.017 | 0 |

^a^ Positive predictive value

^b^ Negative predictive value

^c^ Area under the receiver operating characteristic curve

^d^ Area under the precision-recall curve

^e^ Values for sensitivity, specificity, positive predictive value (PPV), and negative predictive value (NPV) are based on a threshold of 1.298%

^f^ Performance table of original data with probability criterion selected by Youden’s index, optimism corrected performance, and estimated stable optimism

**e-Figure 1:** Receiver operating characteristic curve for fitted LASSO logistic regression predicting post-operative respiratory failure.

This model achieved an observed area under the receiver operating curve (AUC) of 0.854 (95% CI: 0.827 – 0.881) and an optimism-corrected AUC of 0.84 (95% CI: 0.813 – 0.868).


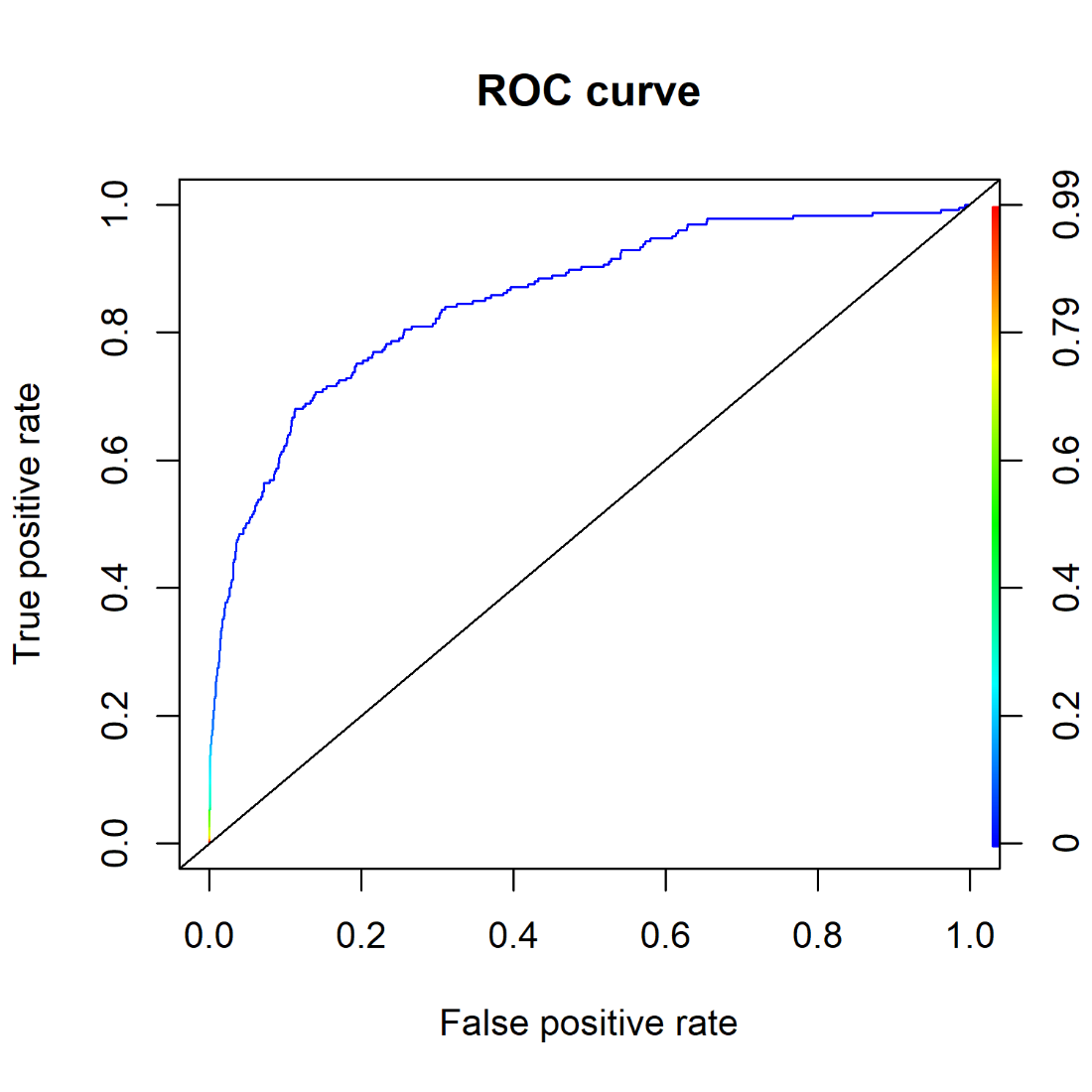


**e-Figure 2:** Precision-recall curve for fitted LASSO logistic regression predicting post-operative respiratory failure.

This model achieved an observed area under the precision-recall curve (AUPRC) of 0.179 (95% CI: 0.127 – 0.227) with an optimism corrected value of 0.162 (95% CI: 0.11 - 0.209).


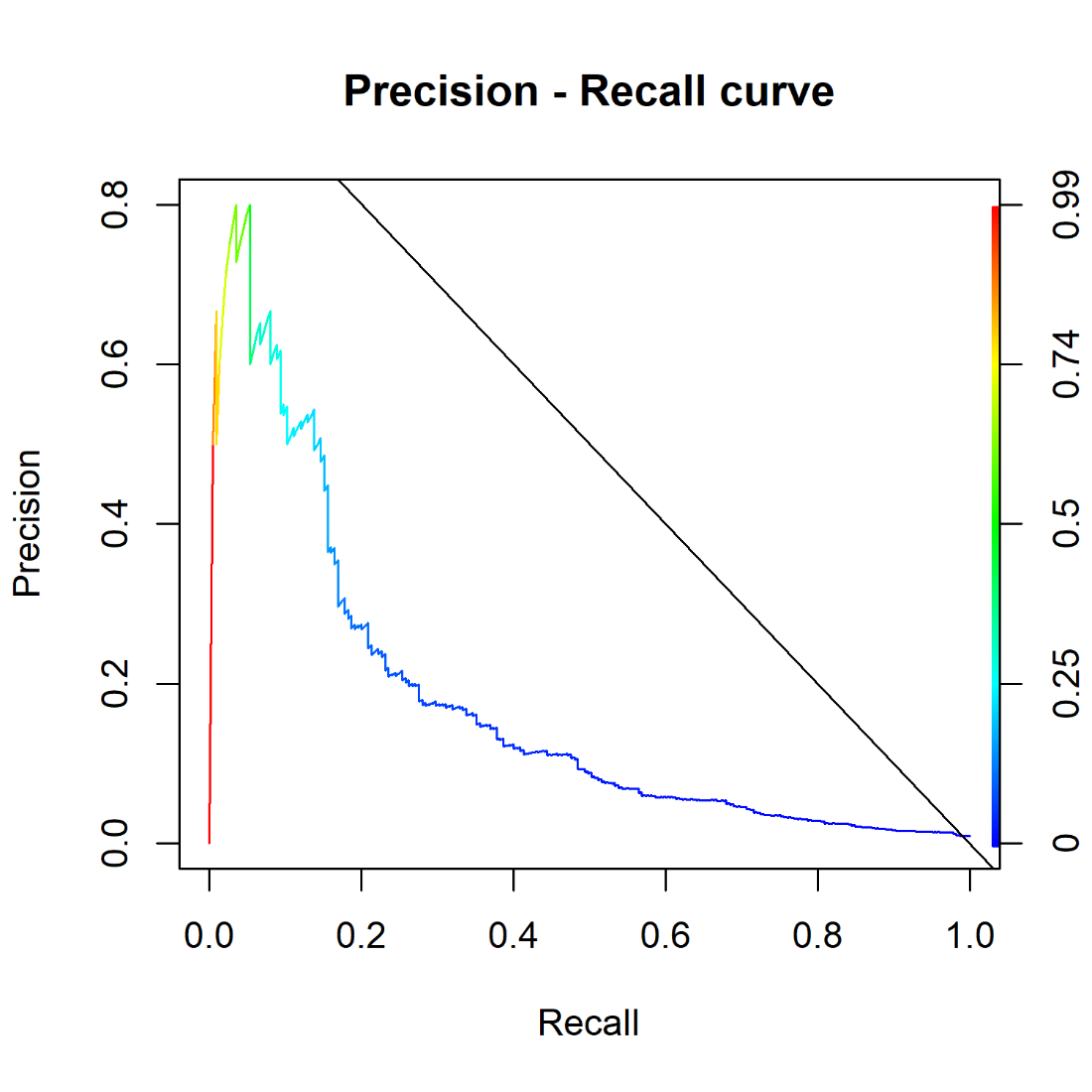


References

1. Leisman DE, Harhay MO, Lederer DJ, et al. Development and Reporting of Prediction Models: Guidance for Authors From Editors of Respiratory, Sleep, and Critical Care Journals. *Critical care medicine*. 2020;48(5):623-633. doi:10.1097/CCM.0000000000004246

2. Moons KG, Altman DG, Reitsma JB, et al. Transparent Reporting of a multivariable prediction model for Individual Prognosis or Diagnosis (TRIPOD): explanation and elaboration. *Annals of internal medicine*. Jan 6 2015;162(1):W1-73. doi:10.7326/m14-0698

3. von Elm E, Altman DG, Egger M, Pocock SJ, Gøtzsche PC, Vandenbroucke JP. The Strengthening the Reporting of Observational Studies in Epidemiology (STROBE) statement: guidelines for reporting observational studies. *Annals of internal medicine*. Oct 16 2007;147(8):573-7. doi:10.7326/0003-4819-147-8-200710160-00010

4. Quan H, Sundararajan V, Halfon P, et al. Coding algorithms for defining comorbidities in ICD-9-CM and ICD-10 administrative data. *Med Care*. Nov 2005;43(11):1130-9. doi:10.1097/01.mlr.0000182534.19832.83

5. van Walraven C, Austin PC, Jennings A, Quan H, Forster AJ. A modification of the Elixhauser comorbidity measures into a point system for hospital death using administrative data. *Med Care*. Jun 2009;47(6):626-33. doi:10.1097/MLR.0b013e31819432e5

6. Kiyatkin ME, Aasman B, Fazzari MJ, et al. Development of an automated, general-purpose prediction tool for postoperative respiratory failure using machine learning: A retrospective cohort study. *Journal of Clinical Anesthesia*. 2023/11/01/ 2023;90:111194. doi:<https://doi.org/10.1016/j.jclinane.2023.111194>

7. Stocking JC, Utter GH, Drake C, et al. Postoperative respiratory failure: An update on the validity of the Agency for Healthcare Research and Quality Patient Safety Indicator 11 in an era of clinical documentation improvement programs. *Am J Surg*. Jul 2020;220(1):222-228. doi:10.1016/j.amjsurg.2019.11.019

8. Stocking JC, Drake C, Aldrich JM, et al. Risk Factors Associated With Early Postoperative Respiratory Failure: A Matched Case-Control Study. *J Surg Res*. Jan 20 2021;261:310-319. doi:10.1016/j.jss.2020.12.043

9. Stocking JC, Drake C, Aldrich JM, et al. Outcomes and risk factors for delayed-onset postoperative respiratory failure: a multi-center case-control study by the University of California Critical Care Research Collaborative (UC3RC). *BMC anesthesiology*. 2022/05/14 2022;22(1):146. doi:10.1186/s12871-022-01681-x

10. Essay P, Mosier J, Subbian V. Rule-Based Cohort Definitions for Acute Respiratory Failure: Electronic Phenotyping Algorithm. *JMIR medical informatics*. Apr 15 2020;8(4):e18402. doi:10.2196/18402

11. Essay P, Mosier J, Subbian V. Phenotyping COVID-19 Patients by Ventilation Therapy: Data Quality Challenges and Cohort Characterization. *Studies in health technology and informatics*. May 27 2021;281:198-202. doi:10.3233/shti210148

12. Essay P, Fisher JM, Mosier JM, Subbian V. Validation of an Electronic Phenotyping Algorithm for Patients With Acute Respiratory Failure. *Crit Care Explor*. Mar 2022;4(3):e0645. doi:10.1097/cce.0000000000000645

13. Peterson TA, Fontil V, Koliwad SK, Patel A, Butte AJ. Quantifying Variation in Treatment Utilization for Type 2 Diabetes Across Five Major University of California Health Systems. *Diabetes Care*. Apr 2021;44(4):908-914. doi:10.2337/dc20-0344

14. Peng Y, Henke E, Reinecke I, Zoch M, Sedlmayr M, Bathelt F. An ETL-process design for data harmonization to participate in international research with German real-world data based on FHIR and OMOP CDM. *International Journal of Medical Informatics*. 2023/01/01/ 2023;169:104925. doi:<https://doi.org/10.1016/j.ijmedinf.2022.104925>

15. Hripcsak G, Duke JD, Shah NH, et al. Observational Health Data Sciences and Informatics (OHDSI): Opportunities for Observational Researchers. *Studies in health technology and informatics*. 2015;216:574-8.
